# Identification of Hypoxia and Mitochondrial-related Gene Signature and Prediction of Prognostic Model in Lung Adenocarcinoma

**DOI:** 10.7150/jca.97374

**Published:** 2024-06-17

**Authors:** Wenhao Zhao, Hua Huang, Zexia Zhao, Chen Ding, Chaoyi Jia, Yingjie Wang, Guannan Wang, Yongwen Li, Hongyu Liu, Jun Chen

**Affiliations:** 1 Department of Lung Cancer Surgery, Tianjin Medical University General Hospital, Tianjin 300052, People's Republic of China.; 2 Tianjin Key Laboratory of Lung Cancer Metastasis and Tumor Microenvironment, Tianjin Lung Cancer Institute, Tianjin Medical University General Hospital, Tianjin 300052, People's Republic of China.

**Keywords:** lung adenocarcinoma, hypoxia, mitochondrial, immune, prognosis.

## Abstract

**Background:** The correlation between hypoxia and tumor development is widely acknowledged. Meanwhile, the foremost organelle affected by hypoxia is mitochondria. This study aims to determine whether they possess prognostic characteristics in lung adenocarcinoma (LUAD). For this purpose, a bioinformatics analysis was conducted to assess hypoxia and mitochondrial scores related genes, resulting in the successful establishment of a prognostic model.

**Methods:** Using the single sample Gene Set Enrichment Analysis algorithm, the hypoxia and mitochondrial scores were computed. Differential expression analysis and weighted correlation network analysis were employed to identify genes associated with hypoxia and mitochondrial scores. Prognosis-related genes were obtained through univariate Cox regression, followed by the establishment of a prognostic model using least absolute shrinkage and selection operator Cox regression. Two independent validation datasets were utilized to verify the accuracy of the prognostic model using receiver operating characteristic and calibration curves. Additionally, a nomogram was employed to illustrate the clinical significance of this study.

**Results:** 318 differentially expressed genes associated with hypoxia and mitochondrial scores were identified for the construction of a prognostic model. The prognostic model based on 16 genes, including PKM, S100A16, RRAS, TUBA4A, PKP3, KCTD12, LPGAT1, ITPRID2, MZT2A, LIFR, PTPRM, LATS2, PDIK1L, GORAB, PCDH7, and CPED1, demonstrates good predictive accuracy for LUAD prognosis. Furthermore, tumor microenvironments analysis and drug sensitivity analysis indicate an association between risk scores and certain immune cells, and a higher risk scores suggesting improved chemotherapy efficacy.

**Conclusion:** The research established a prognostic model consisting of 16 genes, and a nomogram was developed to accurately predict the prognosis of LUAD patients. These findings may contribute to guiding clinical decision-making and treatment selection for patients with LUAD, ultimately leading to improved treatment outcomes.

## Introduction

Lung adenocarcinoma (LUAD) is a type of non-small cell lung cancer. It is characterized by a poor prognosis and a high incidence rate, and has long been a significant challenge to human health 1. Recent research increasingly elucidates the crucial role of genes associated with hypoxia and mitochondria in cancer progression, indicating that they may serve as powerful prognostic indicators.

Hypoxia, defined as inadequate oxygen supply to tissues, commonly arises in solid tumors, due to the chaotic and disorganized structure of tumor vasculature 2, 3. For a long time, it has been widely believed that hypoxia is an effective driving factor for tumor invasiveness, promoting metastasis, cell apoptosis, immune evasion, and treatment resistance 4, 5. In hypoxic environments, tumor cells undergo a series of adaptive changes 6-8.

Mitochondria serve as the energy centers within eukaryotic cells, orchestrating crucial cellular functions including apoptosis, cell differentiation, and information transmission. They achieve this by integrating oxidative phosphorylation, regulating proliferation, and participating in programmed cell death, among other mechanisms 9, 10.

Furthermore, the epigenetic regulation of genes associated with mitochondria significantly influences the initiation, progression, and treatment of tumors 11, 12. Mitochondria serve as the primary sites for oxygen consumption within cells. Consequently, they are significantly impacted by reduced oxygen levels. In response to hypoxic conditions, mitochondria regulate their function through various mechanisms 13. Furthermore, the intermediates of the mitochondrial TCA cycle participate in modulating the hypoxia-inducible factor (HIF), a key regulatory factor in adapting to hypoxia. These studies reveal a complex relationship between hypoxia and mitochondria. Therefore, further research is needed to elucidate how hypoxia and mitochondria interact with each other, as well as their impact on the prognosis of LUAD patients.

Some models have already been established based on hypoxia 14 and mitochondria 15. However, there is currently no research reporting the prognostic impact of combined hypoxia and mitochondrial-related genes in LUAD. To address this gap and expand the therapeutic and diagnostic potential of LUAD, a comprehensive analysis was conducted. In order to enhance reader comprehension of this research, we have constructed a flowchart delineating the key processes (Fig. S1). The goal was to establish a model incorporating hypoxia and mitochondrial-related genes for LUAD.

## Materials and Methods

### Datasets

The gene expression data in Fragments Per Kilobase Million format, along with clinical information for 489 patients with LUAD, as well as gene expression data for 59 adjacent non-cancerous tissues, were all obtained from the The Cancer Genome Atlas Lung Adenocarcinoma (TCGA-LUAD) database. Gene expression data and clinical information for datasets GSE31210 and GSE72094 were retrieved from the Gene Expression Omnibus (GEO) database. The two datasets were built upon the GPL570 and GPL15048 platforms, containing 226 and 393 LUAD samples, respectively. Inclusion criteria comprised complete clinical information and overall survival (OS) greater than 30 days. The training cohort consisted of patients from TCGA-LUAD, while those from GSE31210 and GSE72094 constituted the external validation cohort.

### Single Sample Gene Set Enrichment Analysis (ssGSEA)

The MSigDB was utilized to obtain hypoxia hallmark gene sets, comprising 200 genes (Table S1). As presented in table S2, the 1135 mitochondrial-related genes were extracted from MitoCarta3.0 16. The ssGSEA analysis was conducted for all samples, followed by the calculation of hypoxia and mitochondrial scores for each sample 17.

### Weighted Correlation Network Analysis (WGCNA)

The transcriptome data from TCGA-LUAD were chosen to construct gene coexpression networks utilizing the R package “WGCNA” 18. As indicated in Figure S2, the outlier was identified and subsequently excluded from the analysis. During the network construction phase, a soft thresholding power (β) above 0.90 was determined, demonstrating a fit index for scale-free topology. We established a minimum module size of 30, and utilized the dynamic treecutting algorithm to cluster modules with similar gene expressions, presenting them in a tree diagram with assigned colors. To detect modules linked with hypoxia and mitochondrial scores, we generated a heatmap illustrating module-feature relationships. Modules strongly correlated with both scores were designated as modules of interest, and the genes within these modules were defined as hypoxia and mitochondrial scores-related genes (HMSRGs).

### Identification of DEGs and Functional Enrichment Analysis

The “limma” 19 R package was employed to identify the DEGs in HMSRGs, which were defined as DE-HMSRGs for the subsequent analysis. The "clusterProfiler" 20 R package was utilized to investigate the functions and pathways associated with the DE-HMSRGs through Gene Ontology (GO) and Kyoto Encyclopedia of Genes and Genomes (KEGG) analyses. Moreover, Gene Set Enrichment Analysis (GSEA) was utilized to identify pathways enriched in both groups.

### Construction and validation of a Prediction Model Related to Hypoxia and Mitochondrial Scores

Univariate Cox regression and Least Absolute Shrinkage and Selection Operator (LASSO) Cox regression analyses were conducted to develop a prediction model related to hypoxia and mitochondrial scores, utilizing the R packages “Survival” and “Glmnet” 21. The risk score was determined using the formula: risk score = ∑ (each gene's expression × corresponding coefficient).

### Unsupervised clustering of Hypoxia and Mitochondrial Scores Related model genes

Utilizing the expression profiles of model genes, we employed the "ConsensusClusterPlus" 22 R package to conduct Consensus Clustering (CC) for identifying previously unidentified subtypes of TCGA-LUAD 22. Parameters for CC included selecting "maxK" as 9, "clusterAlg" as "km", and "distance" as "euclidean".

### Construction of a Nomogram for Predicting Survival

A nomogram was developed utilizing the "rms" R package, which incorporated calculated risk scores along with clinical features. Time-dependent Receiver Operating Characteristic (ROC) curves were constructed utilizing the "survivalROC" 23 R package to evaluate the model accuracy. Subsequently, calibration plots were developed using the "rms" R package to assess the predictive accuracy of the nomogram.

### Estimation of Tumor Microenvironments (TME)

ESTIMATE and ssGSEA analyses were employed to quantitatively analyze the levels of immune cells and immune-related pathways. And the expression levels of immune checkpoints between the high-risk and low-risk groups were compared, utilizing the "ggpubr" R package.

### Tumor Mutational Burden (TMB) and Drug Sensitivity Analysis

The TMB for each TCGA-LUAD patient were determined using methods described in previous studies 24. Additionally, the "maftools" 25 package was used to identify the top 20 most mutated genes in TCGA cohort and display the mutation profiles and their frequencies. The distribution of drugs between the groups underwent analysis using the "pRRophetic" 26 R package.

### Statistical Analysis

All statistical analyses were performed using R software (version 4.2.2). Differences between two groups were assessed using either Student's t-test or Wilcoxon test. Survival analysis was depicted using Kaplan-Meier plots and compared using the log-rank test. Statistical significance was defined as P < 0.05.

## Results

### Identification of Hypoxia Score and Mitochondrial Score Related Genes

The ssGSEA analysis was conducted on hypoxia-related genes and mitochondrial-related genes expression profiles within the TCGA-LUAD dataset, aiming to obtain hypoxia and mitochondrial scores. The detailed score results from ssGSEA outputs are presented in Table S3.

**Figure 1:**
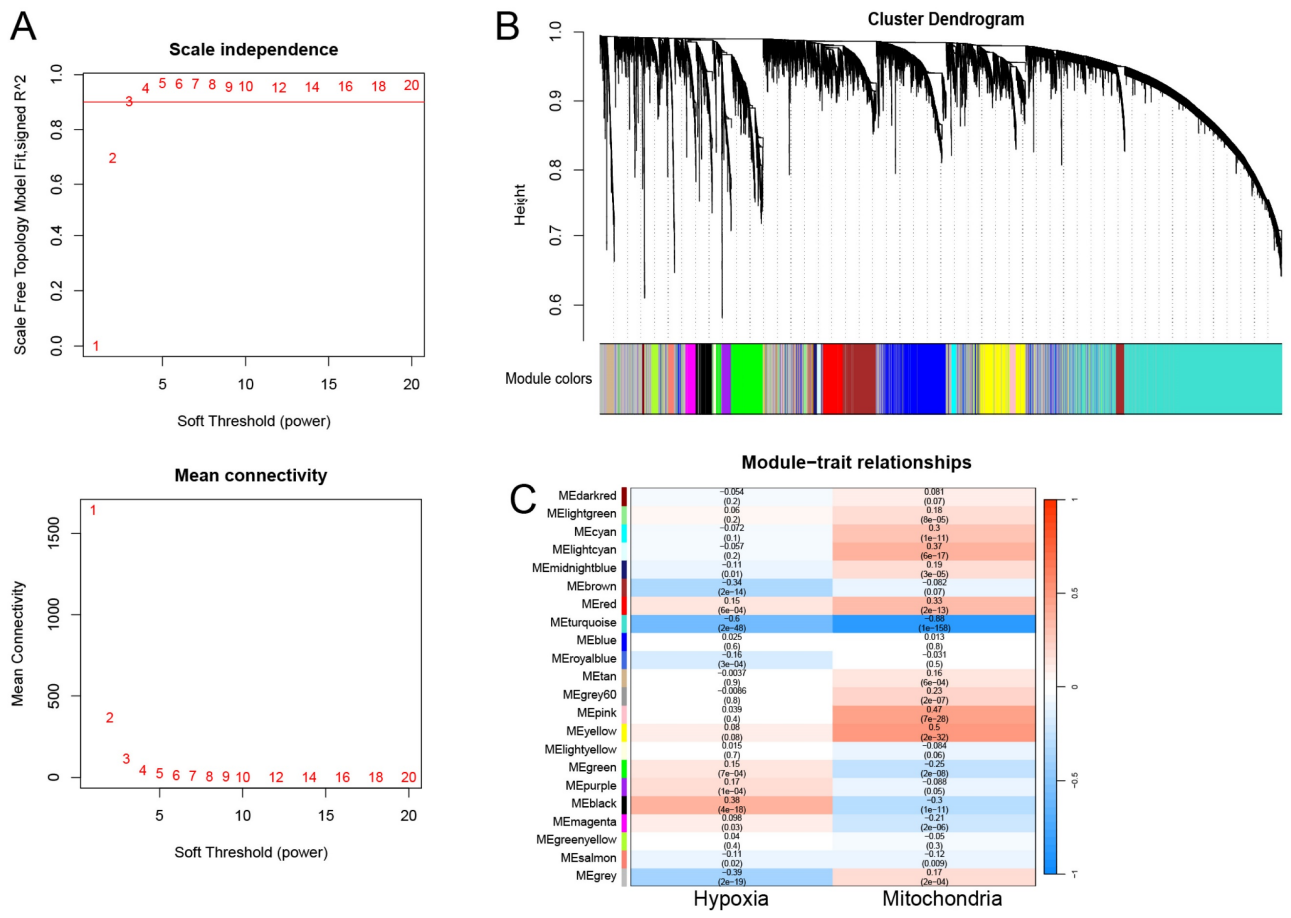
Coexpression Network Construction. (A) The network topology analysis was conducted using various soft threshold powers. (B) Cluster dendrograms of genes based on topological overlap of dissimilarities, and module colors were assigned. (C) Heatmap illustrates the relationship between gene modules and phenotypic traits. The correlation coefficient in each cell reflects this relationship, transitioning from red to blue to indicate decreasing magnitude. The number in parentheses within each cell denotes the correlation P-value.

WGCNA was conducted using the obtained hypoxia and mitochondrial scores as phenotypic data. Following the exclusion of outlier samples, a scale-free network was constructed with a soft threshold parameter set to β=3 (Figure 1A). Finally, 22 modules were identified, each labeled with a different color (Figure 1B). The turquoise module showed the least correlation with the mitochondrial score (cor = -0.88, P = 1e-58) and hypoxia score (cor = -0.6, P = 2e-48) (Figure 1C). Thus, the turquoise module was identified as the module of interest. Overall, a total of 3682 genes were identified as hub genes and designated as HMSRGs, listed in Table S4.

### Identification and Analysis of DE-HMSRGs

**Figure 2:**
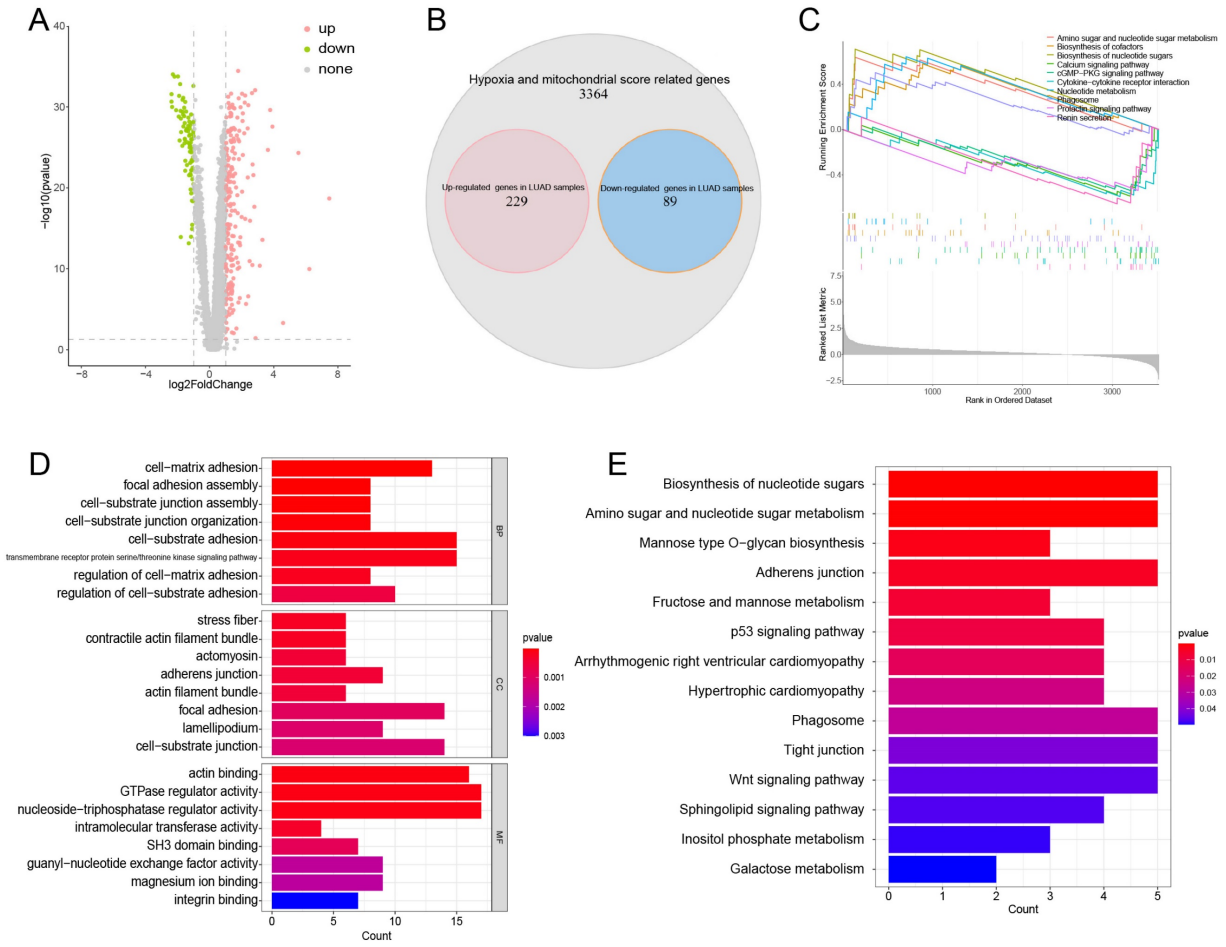
Obtaining and Enrichment analysis for the hypoxia and mitochondrial score related DEGs. (A) Volcano plot showing the DEGs in hub genes between tumor and normal. (B) Venn diagrams showing overlaps of overexpressed genes and hub genes (red: overexpressed genes in tumor samples; blue: overexpressed genes in normal samples). (C) The enriched gene terms in gene set enrichment analysis (GSEA). (D) Column diagrams depicting GO analysis for DEGs related to hypoxia and mitochondrial scores. (E) Column diagrams depicting KEGG analysis for DEGs related to hypoxia and mitochondrial scores.

Differential expression analysis was conducted on 3682 HMSRGs between normal and LUAD samples, a total of 229 up-regulated genes and 89 down-regulated genes were identified in LUAD samples. (Figure 2A, B). GSEA indicates that in LUAD, DE-HMSRGs are primarily enriched in metabolic regulation pathways, while in normal tissues, they are predominantly enriched in various signaling pathways. (Fig. 2C). The GO enrichment analysis indicated that pathways associated with GTPase regulator activity, nucleoside-triphosphatase regulator activity, actin binding, and cell-substrate adhesion (Fig. 2D). KEGG analysis reveals that DE-HMSRGs are predominantly enriched in sugar metabolism and biosynthesis (Fig. 2E).

### Establishment and Validation of a Hypoxia and Mitochondrial Scores Related Prognostic Model

**Figure 3:**
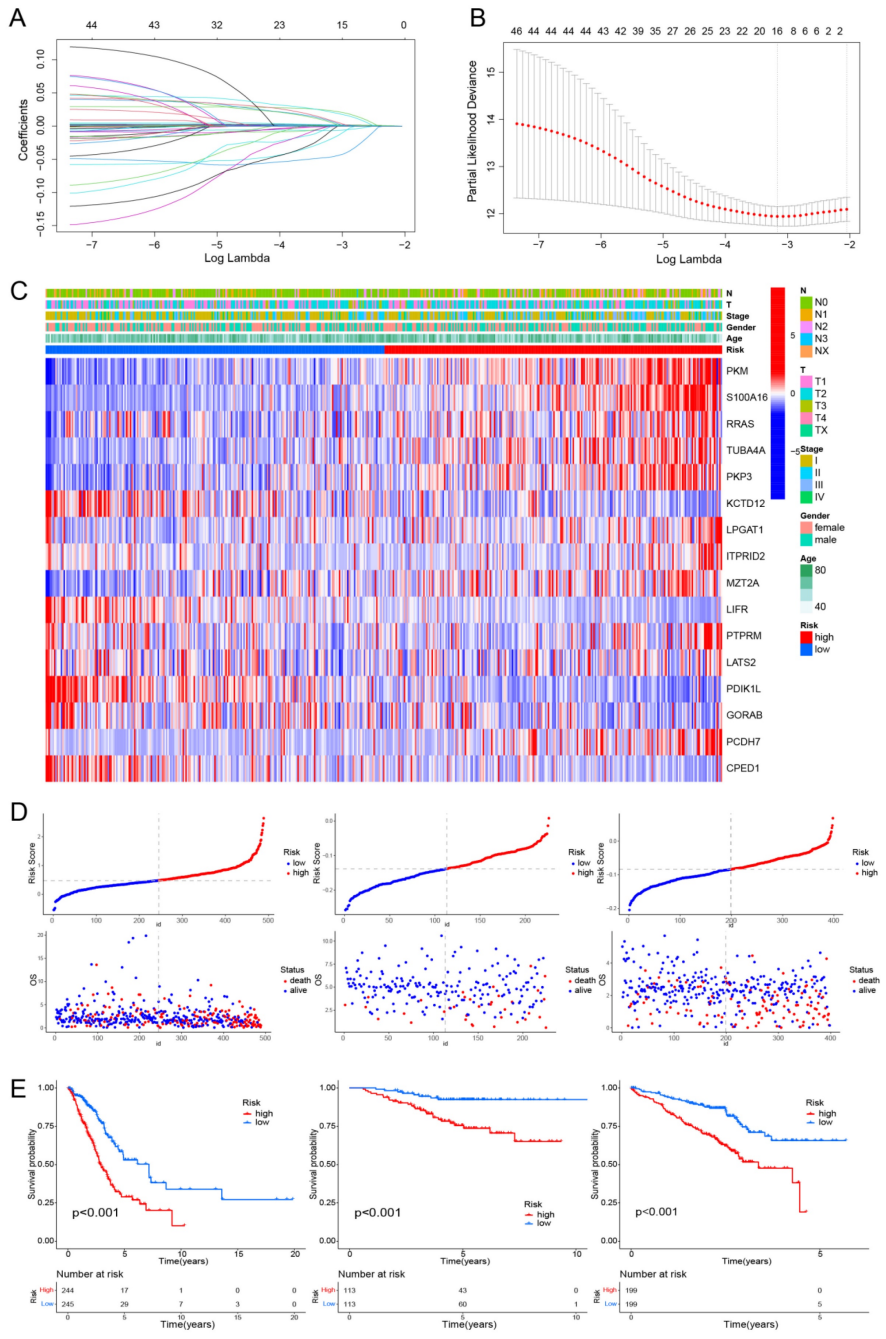
Construction of a hypoxia and mitochondrial score related prognostic model. (A, B) Determining the number of factors using LASSO analysis. (C) Heatmap displaying 16 model genes and clinical features. (D) Distribution of risk score according to the survival status and time in TCGA, GSE31210, and GSE72094 cohorts. (E) Kaplan-Meier curves depicting OS for patients in the different groups.

Using univariate Cox regression and LASSO-Cox regression, a prognostic model was established comprising 16 genes (PKM, S100A16, RRAS, TUBA4A, PKP3, KCTD12, LPGAT1, ITPRID2, MZT2A, LIFR, PTPRM, LATS2, PDIK1L, GORAB, PCDH7, CPED1) (Fig. 3A, B). Table S5 presents the corresponding coefficients of the 16 model genes. The expression levels of PKM, S100A16, RRAS, TUBA4A, PKP3, KCTD12, LPGAT1, ITPRID2, MZT2A, LIFR, PTPRM, LATS2, PDIK1L, GORAB, PCDH7, CPED1 all presented significantly difference between normal and tumor samples (Fig. S3). The LUAD patients were divided into high-risk and low-risk groups based on the median risk score (0.4814). This stratification method was also applied to patients in the validation cohorts GSE31210 and GSE72094. The heatmap indicated T-stage, N-stage, and clinical stage were found to be correlated with the risk scores (Fig. 3C). Figure 3D displays the risk score distributions alongside the survival status. The OS significantly differed between the two patient groups, indicating a worse prognosis for those exhibiting higher risk scores (Fig. 3E). We subsequently conducted subgroup analyses based on clinical features, revealing the strong predictive accuracy of the signature for nearly all LUAD patients prognosis (Fig. S4, 5). The PCA analyses demonstrated favorable results for this prognostic model (Fig. S6). Tables 1, 2, and 3 display the distribution of LUAD patients among various groups based on each clinical feature.

### Unsupervised Clustering of Hypoxia and Mitochondrial Scores Related Model Genes

**Figure 4:**
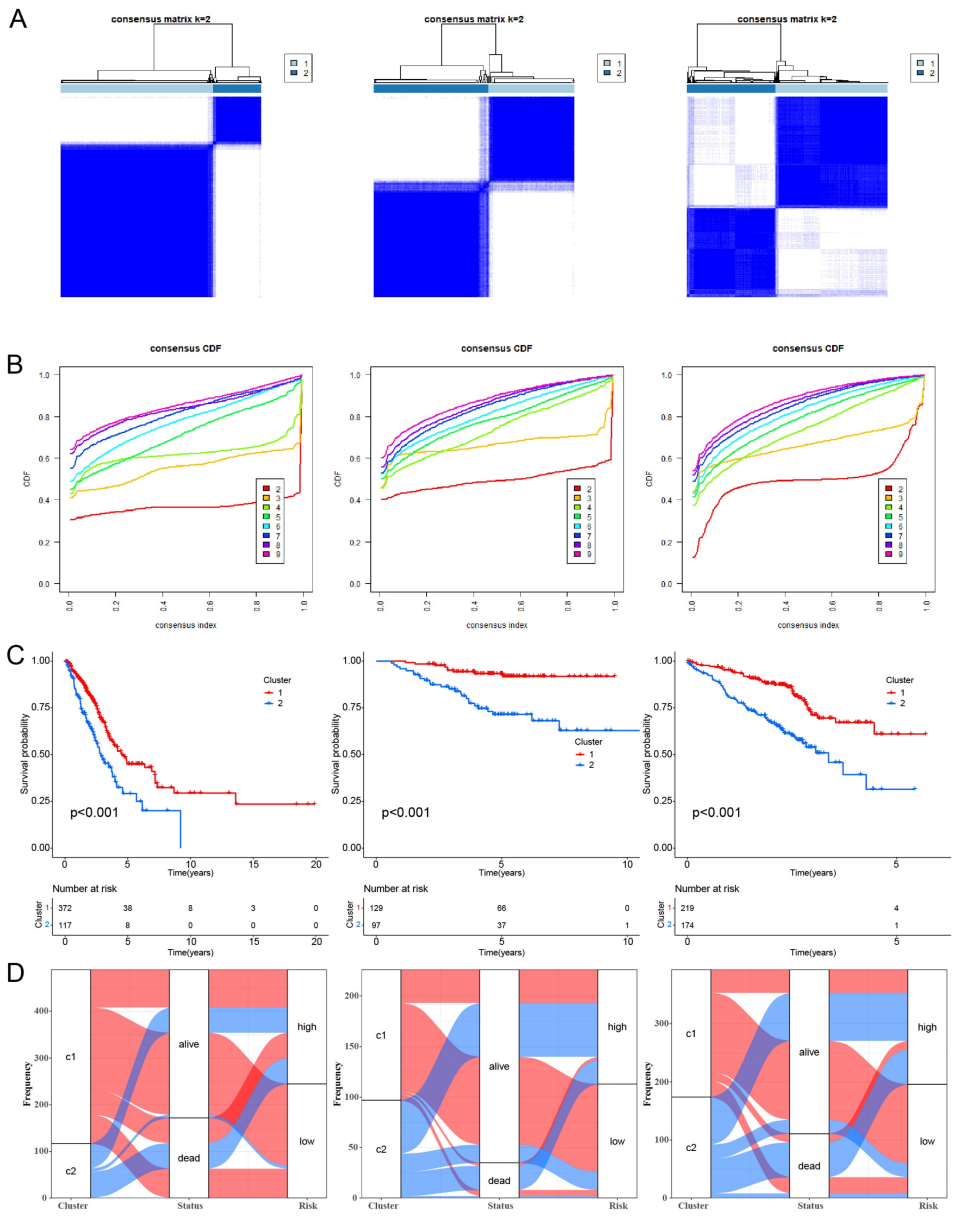
Unsupervised clustering of hypoxia and mitochondrial score related model genes. (A) LUAD patients were grouped into two molecular clusters using a k = 2 approach, relying on the hypoxia and mitochondrial score-related model gene profile. (B) Plotting the empirical cumulative distribution function, we observed consensus distributions for each k value ranging from 2 to 9. (C) Kaplan-Meier analysis of the prognosis of LUAD patients across two distinct molecular clusters. (D) Alluvial diagram illustrates the interrelation among molecular clusters, survival status, and risk groups in LUAD patients.

CC analysis was conducted using the 16 HMSRGs to explore unidentified subtypes within TCGA-LUAD. When K=2, the differences between subgroups are most pronounced, indicating that LUAD can be well distinguished between the two clusters (Fig. 4A, B). The OS between the two clusters showed significant differences (P < 0.001) (Fig. 4C). The alluvial diagrams show that the majority of cluster 1 belongs to the low risk group (Fig. 4D).

### Construction of a Prognostic Nomogram

**Figure 5:**
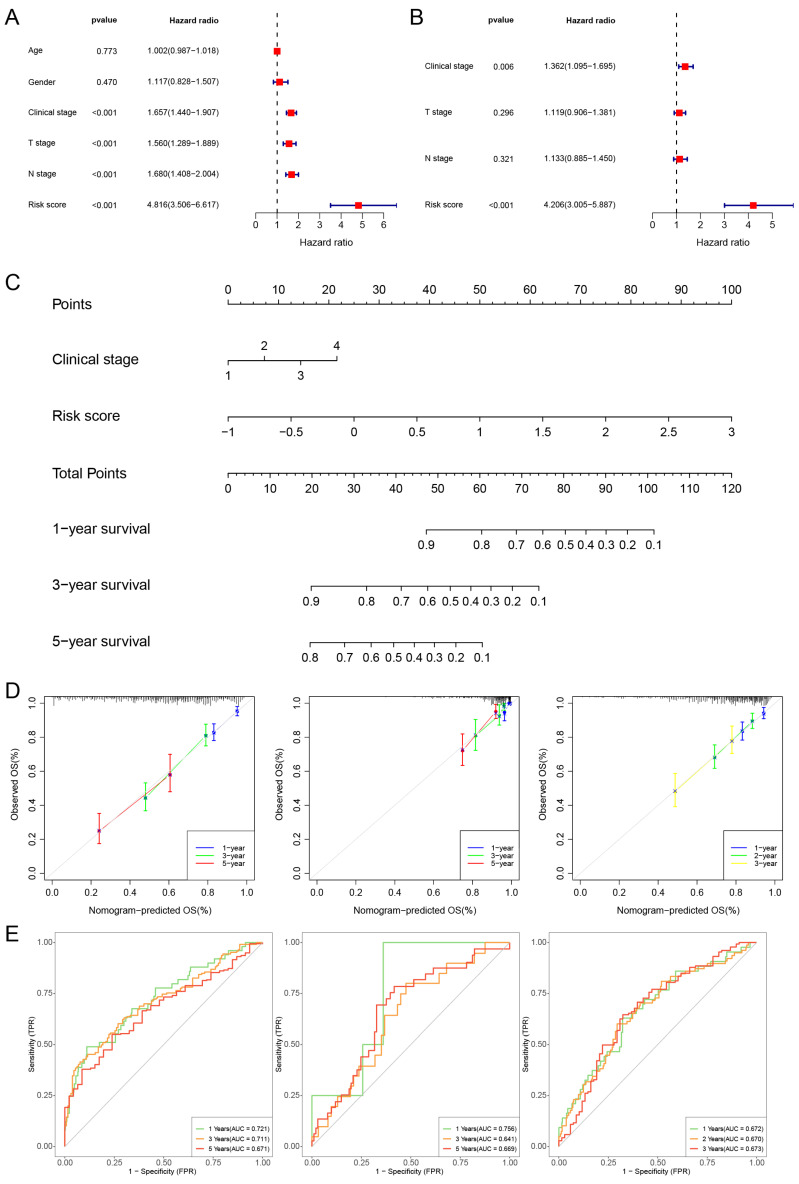
Constructing a nomogram diagram. (A, B) The univariate and multivariate Cox regression analysis of risk score and clinical features. (C) Nomogram of risk score and clinical characteristics (D) Nomogram calibration at 1-, 3-, and 5-years in the TCGA cohort, the GSE31210 cohort, and at 1-, 2-, and 3-years in the GSE72094 cohort. (E) The ROC curve shows the accuracy of the prognostic model.

We then further perform univariate Cox regression and multivariate Cox regression on risk score and clinical features. The findings suggest that the risk score and clinical stage independently influence the prognosis of TCGA-LUAD (Fig. 5A, B). Additionally, we constructed a nomogram and performed calibration, showing its ability to predict the OS rates reasonably well compared to an ideal model in both the TCGA, GSE31210 and GSE72094 cohort (Fig. 5C, D). The ROC curves of the TCGA cohort indicated that the area under the curve (AUC) was 0.721, 0.711, and 0.671 for 1-, 3-, and 5-year OS rates. For the GSE31210 cohort, the AUC values were 0.756, 0.641, and 0.669 for 1-, 3-, and 5-year OS rates, while for the GSE72094 cohort, the AUC values were 0.672, 0.670, and 0.673 for 1-, 2-, and 3-year OS rates, respectively (Fig. 5E).

### TME and Immune Checkpoint Analysis

**Figure 6:**
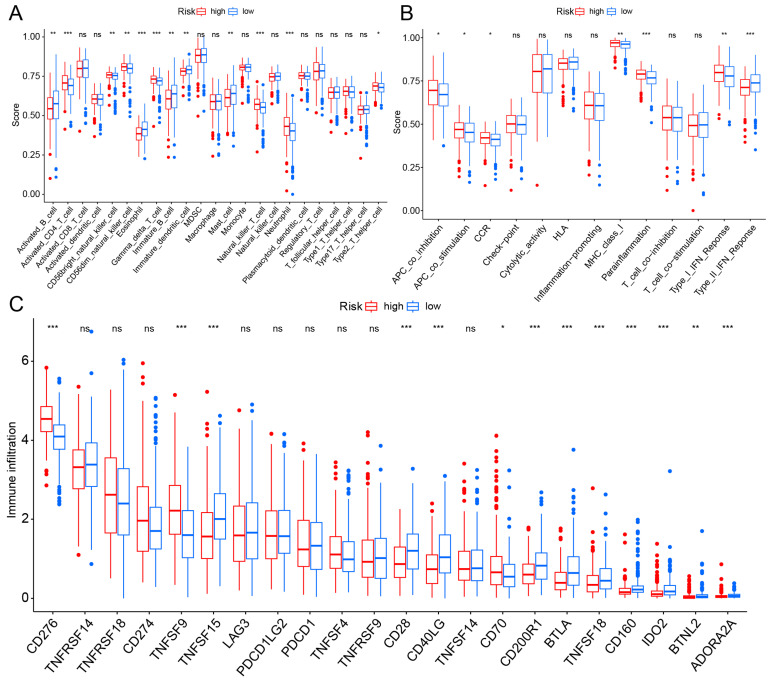
TME and checkpoint analysis. (A) The distribution of 23 immune cell subsets infiltration between two groups. (B) The distribution of 13 immune related pathways between two groups. (C) The distribution of checkpoint related genes between two groups. *p < 0.05, **p < 0.01, ***p < 0.001.

TME is impacted by hypoxia and mitochondria and plays a crucial role in the initiation and progression of cancer. We evaluated the expression levels of infiltrating immune cells and pathways, observing higher expression of activated B cells, eosinophil, immature B cell, and mast cell in the low-risk group. Additionally, pathways with differential expression between the two groups were found to be highly expressed in the high-risk group (Fig. 6A, B). Subsequently, the expression levels of immune checkpoint genes in both groups were compared, revealing that CD276 and TNFSF9 were highly expressed in the high-risk group (Fig. 6C).

### Tumor Mutational Burden (TMB) Analysis

**Figure 7:**
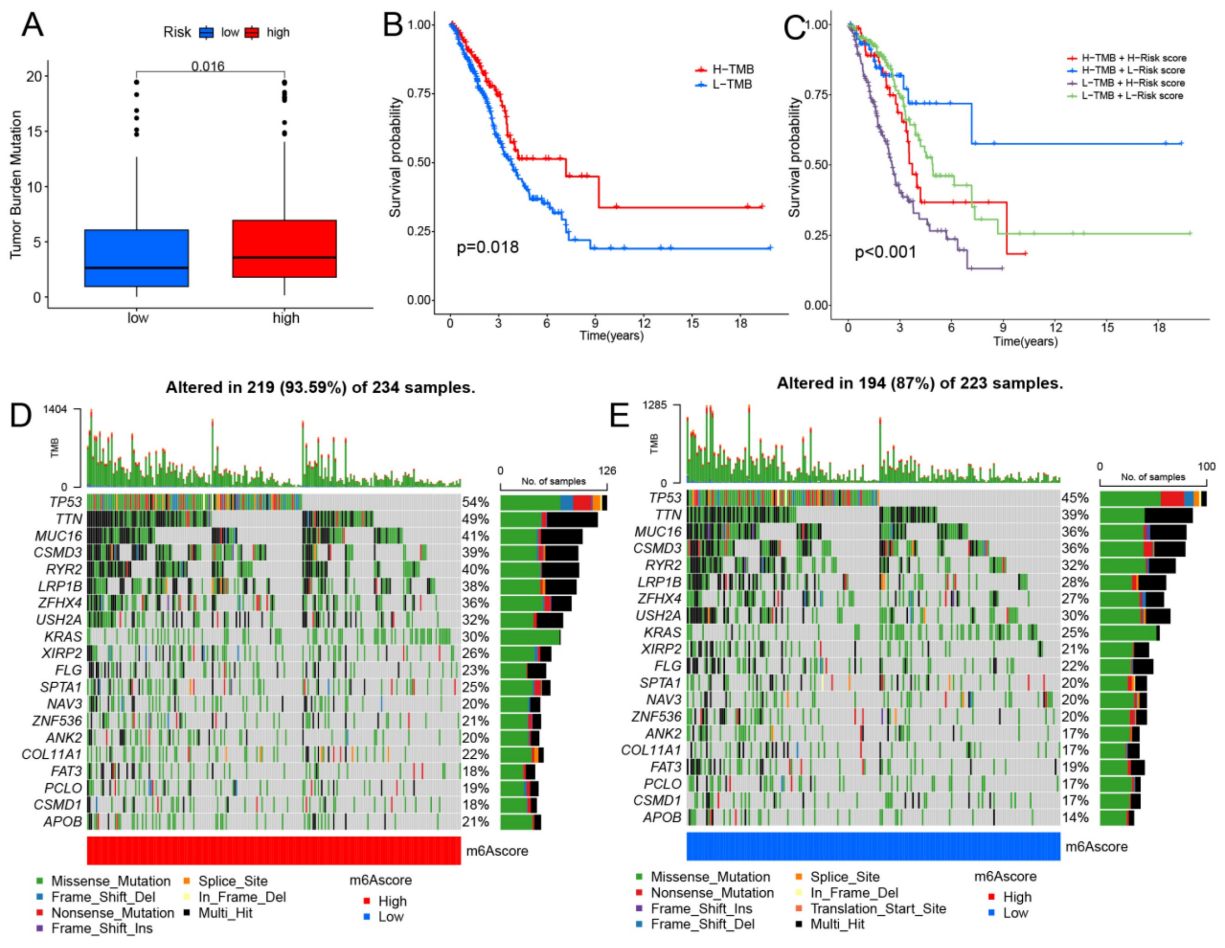
Investigating the correlation between the prognostic model and immunotherapy. (A) The distribution of TMB between two groups. (B) Kaplan-Meier curves depicting OS for patients in the high and low TMB groups. (C) Kaplan-Meier curves illustrate the OS of patients in the combined risk group and TMB group. (D,E) The waterfall plot displays the top 20 mutated genes and their distributional variance in tow groups.

Clinical trials and preclinical studies have shown that immune checkpoint blockade provides long-term clinical benefit, particularly in patients with higher TMB, including improved treatment responses and prolonged OS 27, 28. Results showed that the high-risk group exhibited higher TMB (Fig. 7A). The survival analysis indicates that patients in the high TMB group have better OS (Fig. 7B). Furthermore, the results indicate that patients simultaneously exhibiting high TMB and low-risk scores have the best OS (Fig. 7C). Our results indicate that high-risk patients may exhibit improved treatment responsiveness. Subsequently, we investigated the genetic mutation landscape of TCGA-LUAD, listing the top 20 genes with the highest mutation rates (Fig. 7D, E). The results indicate that the high-risk group exhibits a higher mutation frequency.

### Efficacy of the Model in Predicting Drug Sensitivity

**Figure 8:**
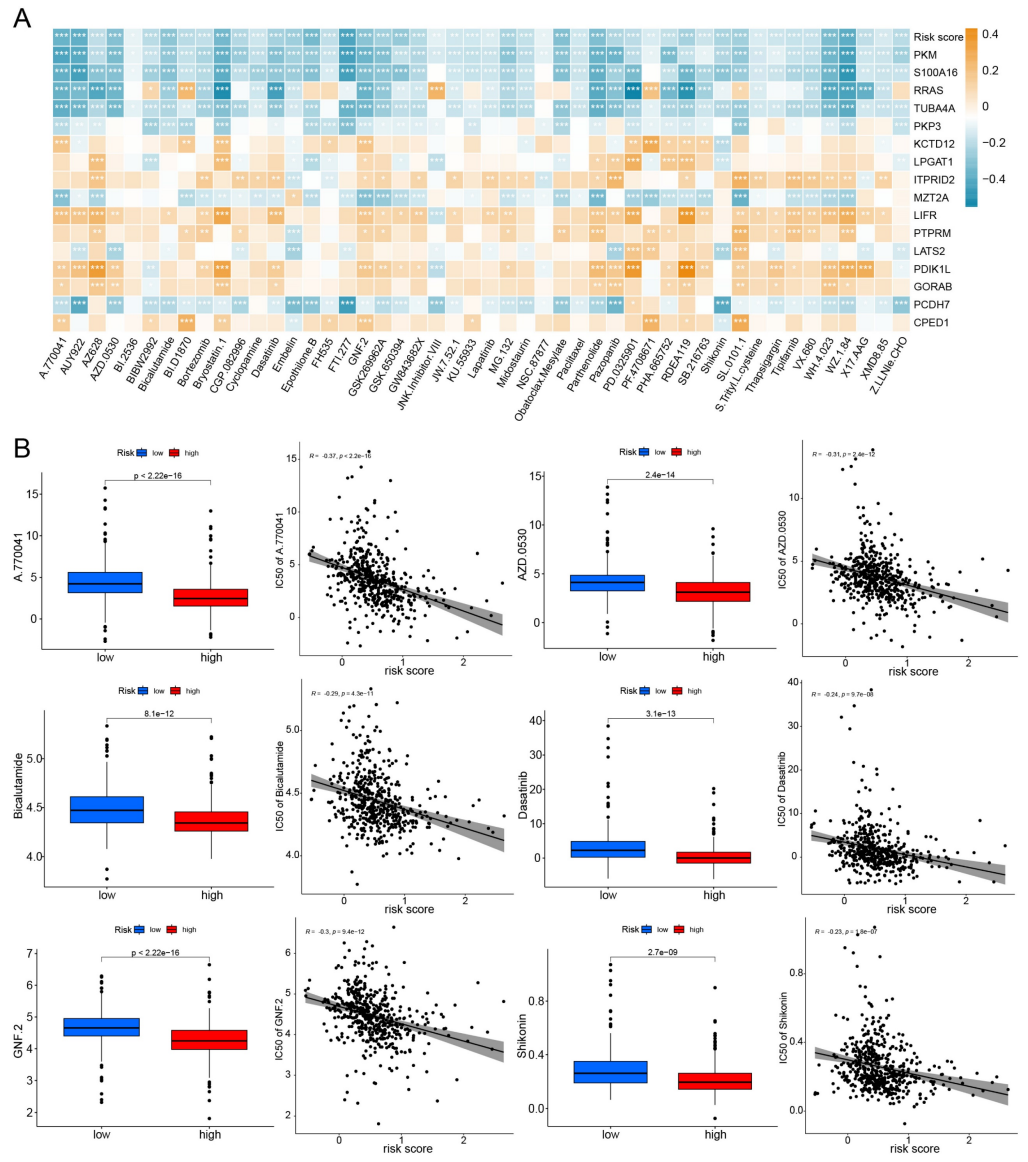
Efficacy of the prognostic model in predicting drug sensitivity. (A) The relationship between drugs, risk score, and model genes; *p < 0.05, **p < 0.01, ***p < 0.001. (B) Boxplots compare the IC50 of drugs between the high-risk and low-risk groups, alongside the correlation between IC50 and risk score.

The half maximal inhibitory concentration (IC50) for each drug was calculated in TCGA-LUAD samples. Correlation analysis was conducted between IC50 and risk scores, selecting 48 drugs with p-values less than 0.01. The relationship between IC50 and model genes was illustrated in Fig. 8A. And box plots were used to illustrate the differences in IC50 for certain drugs between the high-risk and low-risk groups (Fig. 8B). We found that the IC50 values for almost all drugs were lower in the high-risk group. These results suggest that TCGA-LUAD patients with high risk score have higher sensitivity to chemotherapy.

## Discussion

Increasing evidence suggests that the close relationship between hypoxia and mitochondria collectively influences the proliferation and metastasis of LUAD. In this study, we successfully established a prognostic model integrating hypoxia and mitochondrial genes, aiming to better promote the diagnosis and treatment of LUAD.

Based on the expression levels of hypoxia and mitochondrial-related genes, the hypoxia and mitochondrial scores were calculated for each individual sample, respectively. We obtained 22 modules, and the turquoise module was most irrelevant to mitochondrial score (cor = -0.88, P = 1e-58) and hypoxia score (cor = -0.6, P = 2e-48). We then identified 318 different expressed genes from the list of 3682 HMSRGs, which were defined as DE-HMSRGs, respectively.

In this study, key signature genes have been identified in LUAD that exhibit associations with hypoxia and mitochondrial scores, including PKM, S100A16, RRAS, TUBA4A, PKP3, KCTD12, LPGAT1, ITPRID2, MZT2A, LIFR, PTPRM, LATS2, PDIK1L, GORAB, PCDH7, and CPED1, some of these genes has already been reported in previous studies. PKM2 is a key enzyme in the glycolysis process 29, 30. We found that PKM is significantly upregulated in LUAD.

RRAS is involved in invasion and migration 31, 32. We found that RRAS is downregulated in LUAD, and lower expression may correlate with better drug sensitivity. PKP3 is a member of the PKP family. Some studies have confirmed the role of PKP3 in the early occurrence of tumors 33-35. Our research has also found that PKP shows significantly elevated expression in tumor tissues. KCTD12 is involved in forming GABABR. High expression of KCTD12 promotes tumor occurrence by regulating the cell cycle, however, upregulation of KCTD12 inhibits the growth of tumor cells in COAD, UVM, and BRCA 36, 37. Our research found that KCTD12 plays a negative role in the development of LUAD. LIFR has been confirmed to be downregulated in LUAD and hepatocellular carcinoma in previous studies, and it can inhibit tumor invasion and metastasis 38, 39. Our study also confirms this point. GORAB exhibits tumor-suppressive functions in human lung squamous carcinoma cells, as shown in previous reports 40. Our study demonstrates that GORAB similarly exerts this effect in LUAD.

A prognostic model was successfully constructed using the aforementioned 16 genes. And our results indicate the successful establishment of this prognostic model, demonstrating high accuracy. And it was also well demonstrated in the external validation sets GSE31210 and GSE72094 that this model exhibits high accuracy. Compared to other similar prognosis models, our prognosis model possesses higher predictive accuracy. Liu et al. established a prognostic model based on hypoxia-associated genes, with AUC values of 0.66, 0.72, and 0.62 for 1-, 3-, and 5-year survival rates, respectively 14. Yang et al. developed a new hypoxia-related prognostic risk score model, and the AUC values of this model were 0.70, 0.67, and 0.68, respectively, for 1-, 3-, and 5-year OS 41. Subsequently, a nomogram was constructed by integrating the prognostic model with clinical features. We subsequently assessed the correlation between immune checkpoints and the prognostic model, identifying immune checkpoints with significant expression differences between the two groups, including CD276, TNFSF9, TNFSF15, CD28, CD40LG, among others. This may offer new insights into the treatment of LUAD. Next, we investigated the relationship between the prognostic model and TMB, revealing significant differences in TMB between the high and low-risk groups, suggesting that our model may guide immunotherapy for LUAD. Finally, drug sensitivity analysis showed that the majority of drugs had lower IC50 values in the high-risk group. This phenomenon can be explained by the fact that high-risk patients typically have a higher tumor mutation burden, making them more likely to respond positively to immunotherapy and targeted therapy, indicating that TCGA-LUAD patients with high risk scores may be sensitive to standard chemotherapy regimens.

However, this study has several limitations. Due to the limited number of patients with the OS exceeding 5 years in the GSE72094 dataset, we only conducted analyses related to 1-, 2-, and 3-year survival for this dataset. Although the utilization of sound bioinformatics methods and validation across multiple databases has significantly facilitated the establishment of prognostic models for LUAD 42, it is important to note that most studies to date have relied on gene expression data from various databases, lacking detailed insights into underlying biological processes. Moreover, the prognostic model we have developed may inherently exhibit some bias because we cannot account for the intrinsic variations within tumors, such as differences in hypoxia within and outside the tumor. Additionally, all data in this study are retrospective, emphasizing the critical need for experimental research to validate the conclusions drawn in this article.

In conclusion, we have developed a prognostic model with high predictive accuracy for forecasting the OS of LUAD. The prognostic model can predict the prognosis of LUAD patients well. Additionally, it contributes to the study of immune infiltration in the immune microenvironment of LUAD and provides new insights into the comprehensive treatment plan for LUAD patients.

## Conclusions

In conclusion, the study contributes to the enhanced comprehension of LUAD by underscoring the crucial role of hypoxia and mitochondrial scores related genes and presenting a pragmatic prognostic model for clinical application. This model has the potential to stratify risk and tailor highly individualized treatment plans for LUAD patients, thereby improving their prognosis. Further research in this direction may contribute to the development of more effective treatment strategies and advance the diagnosis and treatment of LUAD.

## Supplementary Material

Supplementary figures and tables.

## Figures and Tables

**Table 1:** The association of clinicopathological features in TCGA cohort.

TCGA-LUAD cohort			
Characteristics	High risk (%)	Low risk (%)	P value
Age			
≤65 year	125 (51.2%)	118 (48.2%)	0.498
>65 year	119 (48.8%)	127 (51.8%)	
Gender (%)			
Female	124 (50.8%)	138 (56.3%)	0.222
Male	120 (49.2%)	107 (43.7%)	
Pathologic stage			
Ⅰ	120 (49.2%)	152 (62.0%)	0.004
Ⅱ	60 (24.6%)	57 (23.3%)	
Ⅲ	51 (20.9%)	24 (9.8%)	
Ⅳ	13 (5.3%)	12 (4.9%)	
T stage			0.224
T1	72 (29.5%)	96 (39.2%)	
T2	137 (56.1%)	120 (49.0%)	
T3	25 (10.2%)	20 (8.2%)	
T4	9 (3.7%)	7 (2.9%)	
TX	1 (0.4%)	2 (0.8%)	
N stage			<0.001
N0	142 (58.2%)	181 (73.9%)	
N1	54 (22.1%)	35 (14.3%)	
N2	44 (18.0%)	20 (8.2%)	
N3	1 (0.4%)	1 (0.4%)	
Nx	3 (1.2%)	8 (3.3%)	
M stage			0.837
M0	164 (67.2%)	162 (66.1%)	
M1	13 (5.3%)	11 (4.5%)	
Mx	67 (27.5%)	72 (29.4%)	

**Table 2:** The association of clinicopathological features in GSE31210 cohort.

GSE31210 cohort			
Characteristics	High risk (%)	Low risk (%)	P value
Age			
≤65 year	88 (77.9%)	88 (77.9%)	1
>65 year	25 (22.1%)	25 (22.1%)	
Gender (%)			
Female	48 (42.5%)	73 (64.6%)	0.001
Male	65 (57.5%)	40 (35.4%)	
Pathologic stage			
Ⅰ	69 (61.1%)	99 (87.6%)	<0.001
Ⅱ	44 (38.9%)	14 (12.4%)	

**Table 3:** The association of clinicopathological features in GSE72094 cohort.

GSE72094 cohort			
Characteristics	High risk (%)	Low risk (%)	P value
Age			
≤65 year	58 (29.6%)	59 (29.9%)	0.938
>65 year	138 (70.4%)	138 (70.1%)	
Gender (%)			
Female	98 (50.0%)	121 (61.4%)	0.023
Male	98 (50.0%)	76 (38.6%)	
Pathologic stage			
Ⅰ	114 (58.2%)	140 (71.1%)	0.022
Ⅱ	41 (20.9%)	26 (13.2%)	
Ⅲ	35 (17.9%)	22 (11.2%)	
Ⅳ	6 (3.1%)	9 (4.6%)	
