# Supplementary material for: Identification of Hypoxia and Mitochondrial-related Gene Signature and Prediction of Prognostic Model in Lung Adenocarcinoma
Source: J Cancer. 2024 Jun 17;15(14):4513–26. doi: 10.7150/jca.97374 (PMC11242342; doi:10.7150/jca.97374)
Supplement: Supplementary file 1 — Supplementary figures and tables. [file jcav15p4513s1.pdf]

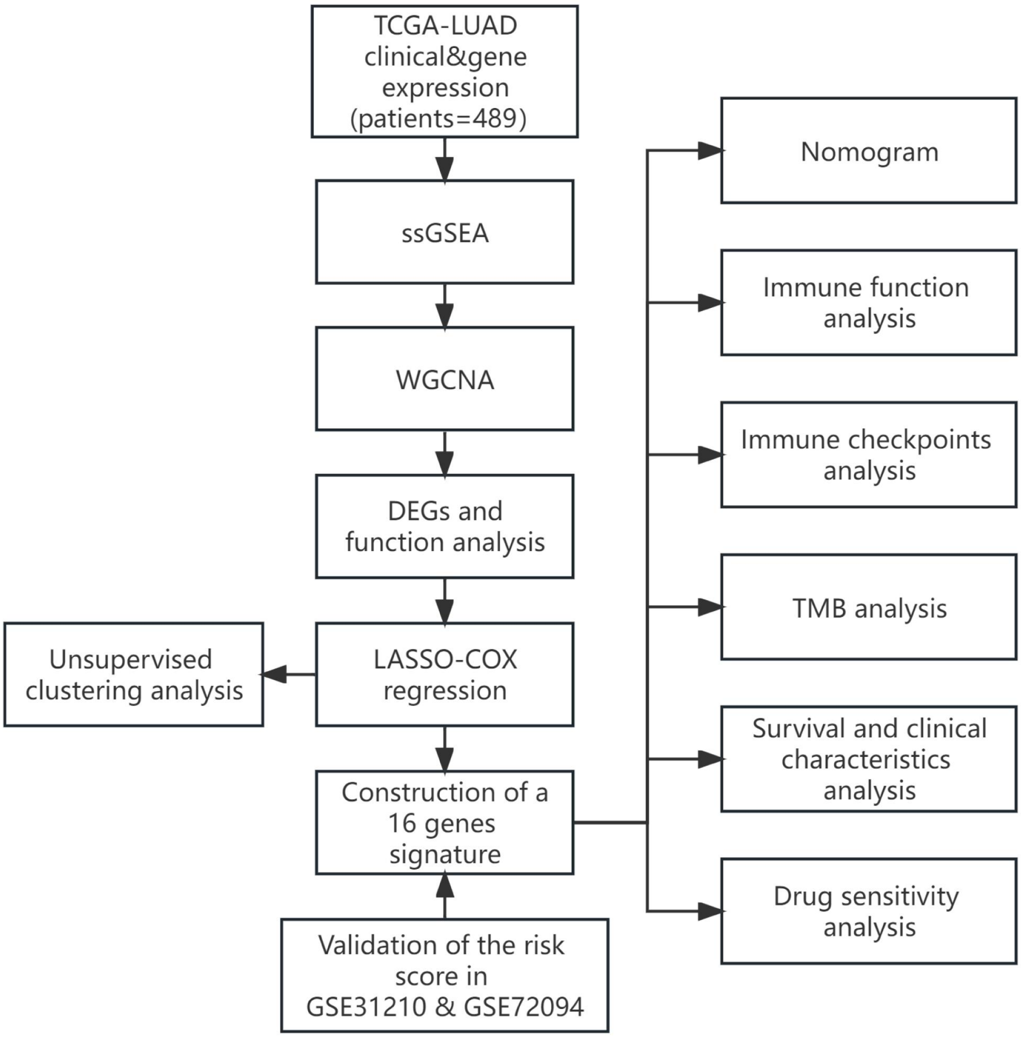

Fig. S1
The entire analytical process of the study.

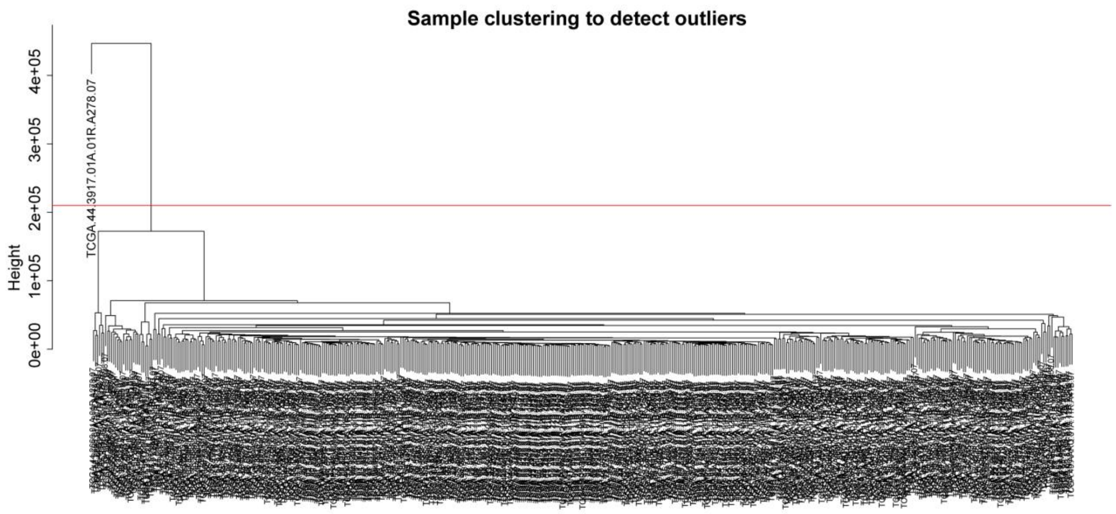

Fig. S2
cluster analysis shows that TCGA-44-3917-01A-01R-A278-07 was identified as an outlier.

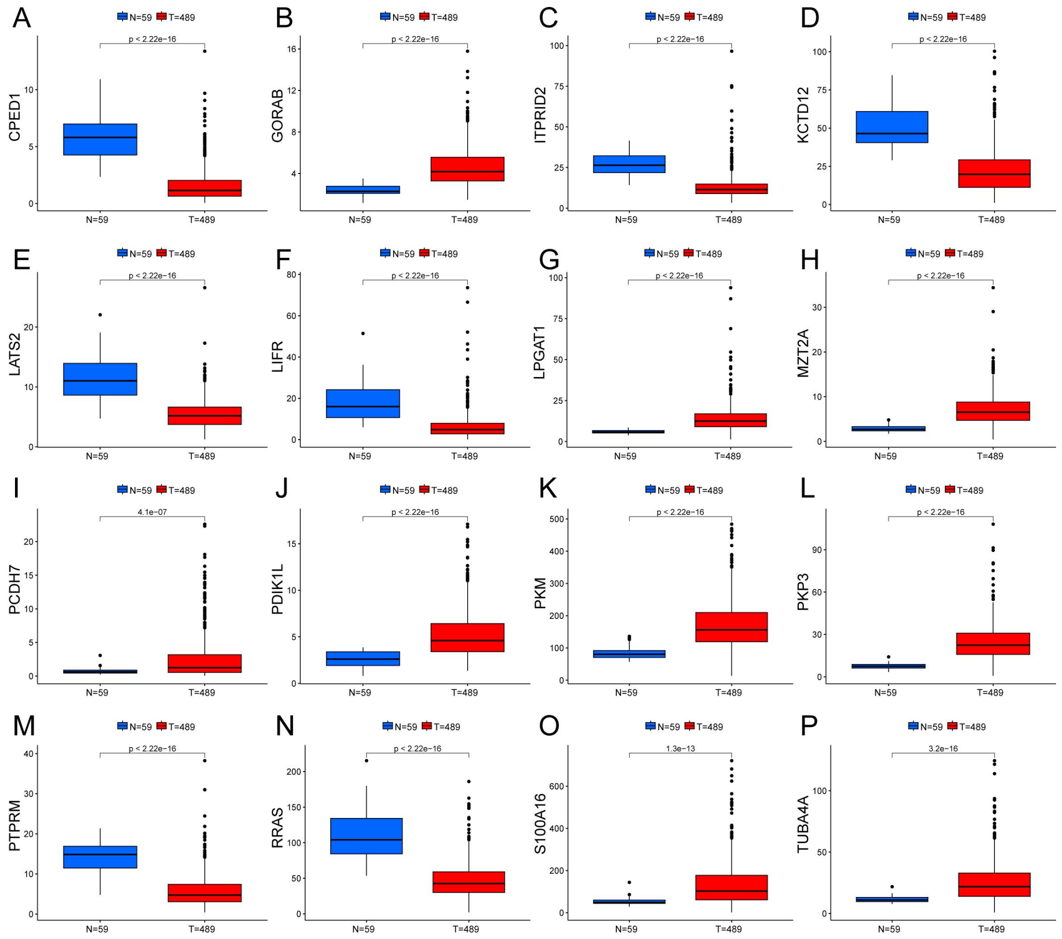

Fig. S3

The expression levels of (A) CPED1, (B) GORAB, (C) ITPRID2, (D) KCTD12, (E) LATS2, (F) LIFR, (G) LPGAT1, (H) MZT2A, (I) PCDH7, (J) PDIK1L, (K) PKM, (L) PKP3, (M) PTPRM, (N) RRAS, (O) S100A16, and (N) TUBA4A in tumor tissues and normal tissues.

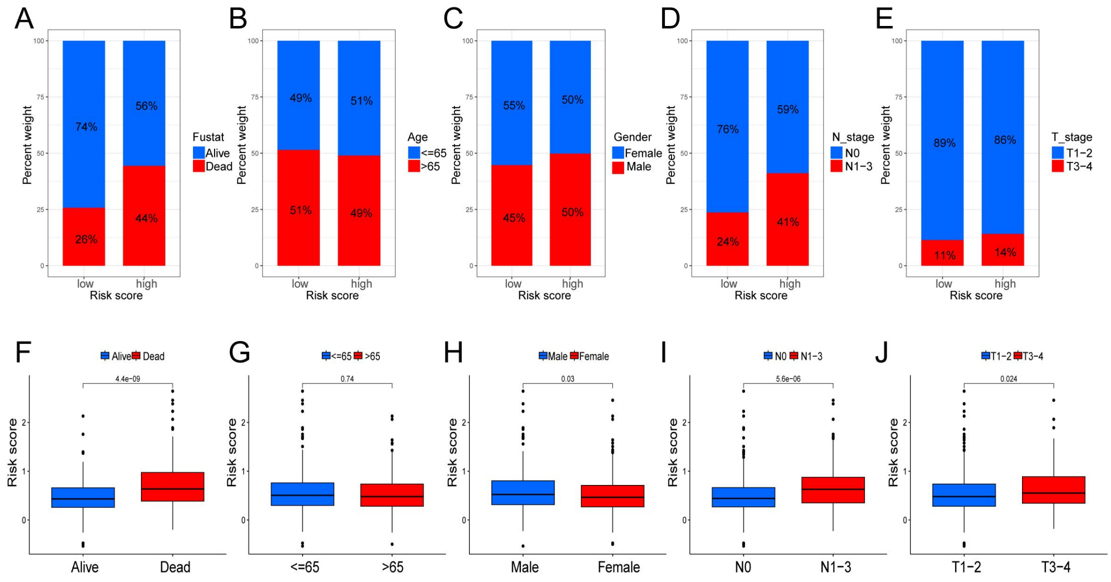

Fig. S4

Clinical subgroup analysis encompassing clinicopathological characteristics. (A-E) The percentage of clinicopathological characteristics in low risk and high risk groups. (F-J) Boxplots show the differences in the risk score between clinical subgroups.

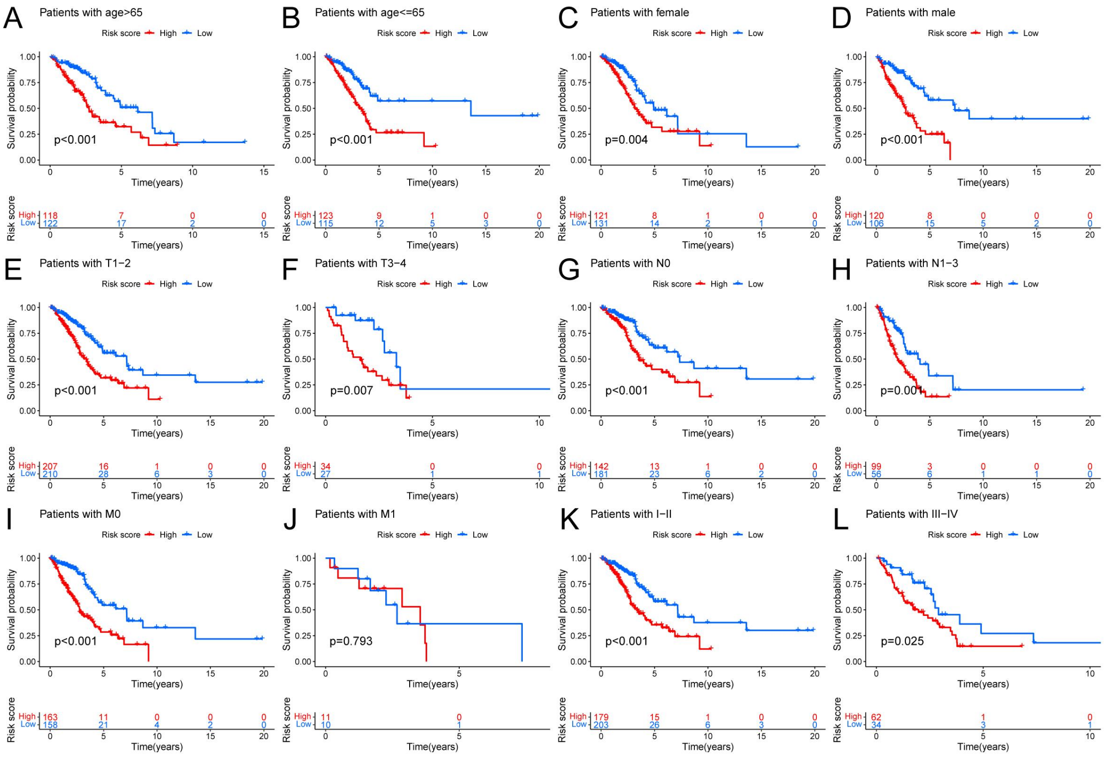

Fig. S5

Kaplan–Meier plots of overall survival for patients in the high risk and low risk groups within (A, B) age, (C, D) gender, (E, F) T stage, (G, H) N stage, (I, J) M stage, (K, L) clinical stage subgroups.

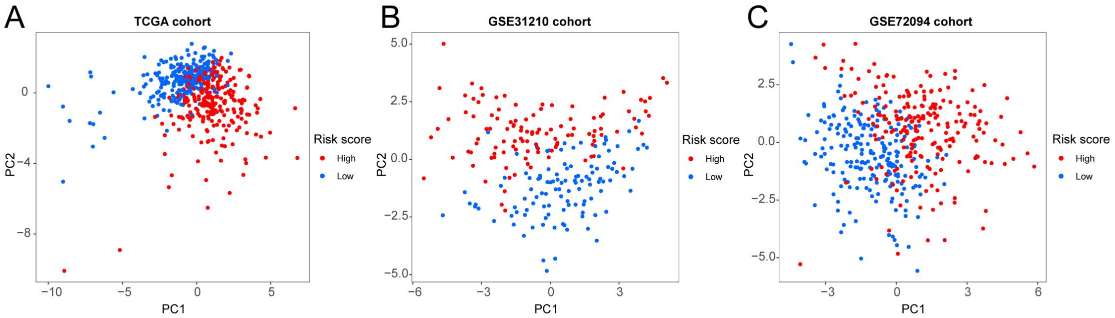

Fig. S6

PCA analysis demonstrating significant differences between high risk and low risk patients in the (A) TCGA cohort, (B) GSE31210 cohort, and (C) GSE72094 cohort.

Table S1. Summary of hypoxia-related genes.

Hypoxia-related genes				
ADM	DCN	HK1	NDST2	SAP30
ADORA2B	DDIT3	HK2	NEDD4L	SCARB1
AK4	DDIT4	HMOX1	NFIL3	SDC2
AKAP12	DPYSL4	HOXB9	NR3C1	SDC3
ALDOA	DTNA	HS3ST1	P4HA1	SDC4
ALDOB	DUSP1	HSPA5	P4HA2	SELENBP1
ALDOC	EDN2	IDS	PAM	SERPINE1
AMPD3	EFNA1	IER3	PCK1	SIAH2
ANGPTL4	EFNA3	IGFBP1	PDGFB	SLC25A1
ANKZF1	EGFR	IGFBP3	PDK1	SLC2A1
ANXA2	ENO1	IL6	PDK3	SLC2A3
ATF3	ENO2	ILVBL	PFKFB3	SLC2A5
ATP7A	ENO3	INHA	PFKL	SLC37A4
B3GALT6	ERO1A	IRS2	PFKP	SLC6A6
B4GALNT2	ERRFI1	ISG20	PGAM2	SRPX
BCAN	ETS1	JMJD6	PGF	STBD1
BCL2	EXT1	JUN	PGK1	STC1
BGN	F3	KDELR3	PGM1	STC2
BHLHE40	FAM162A	KDM3A	PGM2	SULT2B1
BNIP3L	FBP1	KIF5A	PHKG1	TES
BRS3	FOS	KLF6	PIM1	TGFB3
BTG1	FOSL2	KLF7	PKLR	TGFBI
CA12	FOXO3	KLHL24	PKP1	TGM2
CASP6	GAA	LALBA	PLAC8	TIPARP
CAV1	GALK1	LARGE1	PLAUR	TKTL1
CCNG2	GAPDH	LDHA	PLIN2	TMEM45A
NOCT	GAPDHS	LDHC	PNRC1	TNFAIP3
CDKN1A	GBE1	LOX	PPARGC1A	TPBG
CDKN1B	GCK	LXN	PPFIA4	TPD52
CDKN1C	GCNT2	MAFF	PPP1R15A	TPI1
CHST2	GLRX	MAP3K1	PPP1R3C	TPST2
CHST3	GPC1	MIF	PRDX5	UGP2
CITED2	GPC3	MT1E	PRKCA	VEGFA
COL5A1	GPC4	MT2A	CAVIN3	VHL
CP	GPI	MXI1	CAVIN1	VLDLR
CSRP2	GRHPR	MYH9	PYGM	CCN5
CCN2	GYS1	NAGK	RBPJ	WSB1
CXCR4	HAS1	NCAN	RORA	XPNPEP1
ACKR3	HDLBP	NDRG1	RRAGD	ZFP36
CCN1	HEXA	NDST1	S100A4	ZNF292

Table S2. Summary of mitochondrial-related genes.

Mitochondrial-related genes				
MT-CO1	COX6A1	TFAM	GCDH	TEFM
MT-CO3	ATP5MC1	DNM1L	ATPSCKMT	COX16
MT-CO2	NDUFA12	PPTC7	NADK2	SETD9
MT-ND4	NBR1	MPV17	LDHD	ATP23
MT-ATP6	ALDH2	MRPL13	EARS2	NUBPL
MT-CYB	MPC1	MRPS18A	MIPEP	PCBD2
MT-ND2	ATP5F1D	UQCC2	NIT2	NSUN3
MT-ND1	MRPS12	GRHPR	TAZ	RTN4IP1
MT-ND3	STYXL1	MRPS27	FOXRED1	LARS2
MT-ND6	DGUOK	PRKACA	FDXR	FAM185A
MT-ND4L	NRDC	ECHDC3	DHRS7B	TMEM143
MT-ND5	MRPL55	DNAJA3	GFM1	ADCK1
MT-ATP8	TRIAP1	TRAP1	D2HGDH	LIPT1
SLC25A6	AKAP1	GRPEL1	NDUFA5	L2HGDH
PRDX5	LONP1	LETMD1	ALDH5A1	ACSM3
GPX1	BOK	QDPR	AGPAT5	NDUFB8
CHCHD2	TOMM40	TRMT1	NLRX1	MTFR2
ATP5F1B	PAICS	REXO2	MTFR1L	PAM16
NDUFS5	TIMM17A	MTFR1	MTRES1	SUGCT
SLC25A5	MICU1	ABHD10	C2orf69	METTL15
NDUFA1	SDHD	FAHD1	NGRN	MTARC1
GPX4	MRPS7	TIMM50	AARS2	BCL2
PRDX6	COX7A2L	NIT1	SLC25A25	ALDH1L2
COX6B1	SCP2	MRPS2	CHCHD5	BOLA3
NDUFB7	MRPL34	MRPL23	MRM3	ACSF3
COX7C	MRPL57	ACAD8	TMEM177	OGG1
ECHS1	ISCU	BCL2L2	OSBPL1A	PLD6
MCL1	UQCRFS1	HMGCL	MECR	METAP1D
COX8A	MRPL10	ELAC2	FASTKD2	METTL8
NDUFB11	NDUFS8	CLPX	ABCB8	CMC1
STOM	FPGS	ATPAF1	ME3	SFXN5
SOD1	NSUN2	PDHB	ABCB7	RDH13
TUFM	MRPL28	MRPS6	ANGEL2	SLC25A45
ROMO1	NIPSNAP2	COA6	NDUFA10	GATB
MRPL14	BCKDK	ADCK2	TMEM65	NDUFA11
TMEM14C	TIMM17B	MRPS25	HADH	SPRYD4
FKBP8	TOMM70	BIK	CMPK2	HEMK1
TSPO	NDUFB1	CISD1	SLC25A16	MTO1
GHITM	AK2	PNPLA8	CCDC90B	NUDT13
UQCRH	UNG	ATAD3A	SPIRE1	NDUFAF5
APEX1	MRPL11	ACAA2	AASS	NT5M

TOMM20	NAXD	PDHX	RPUSD3	EXOG
PARK7	SQOR	MAIP1	PRODH	SLC25A27
IFI27	LRPPRC	RSAD1	ZADH2	CPT2
NDUFS6	TMEM126A	MACROD1	FASTKD3	ENDOG
TOMM7	MRPL9	COX7A1	COQ7	HPDL
PRDX4	BNIP3	ARMC10	TRMT61B	AGMAT
GOLPH3	MRPL44	MRPL35	TMEM186	RBFA
MTCH1	COMT	CCDC58	PUSL1	MMAA
NAXE	MPC2	ACAD9	SLC25A40	TAMM41
UCP2	ATP5MPL	ISCA1	SIRT3	MTHFD2L
TXNRD1	GTPBP6	BCL2L13	NDUFAF4	MTRF1L
COX5B	SUCLG1	QTRT1	PUS1	RAB24
ATP5F1C	NDUFA3	PMPCA	WARS2	SIRT4
NDUFB10	MUL1	SLC25A4	BCKDHB	DHRS2
VDAC1	SMIM20	SDSL	STX17	TMLHE
CYC1	SLC25A23	TTC19	COA1	LIPT2
MDH2	GLRX5	C8orf82	GATC	MICOS10
IDH2	MRPL16	PNPT1	GRPEL2	SARDH
ATP5MC2	NDUFA2	ATP5ME	MRPL22	SDR39U1
COX5A	MRPL40	MFN1	TXNRD2	RAB5IF
HSD17B10	RHOT2	GARS1	ALKBH1	PRELID3A
SLC25A39	FAM136A	MRS2	CARS2	MPV17L
MRPL51	HSDL2	ARMCX2	MMAB	NDUFA9
ACADVL	PLPBP	NIPSNAP3A	COQ3	IBA57
HIGD2A	RPIA	HARS2	MIEF2	ACACB
ATP5MD	ATP5MF	TIMM29	THEM4	SERAC1
MRPS34	HDHD3	OPA1	ATAD3B	CKMT1B
AKR1B10	NFU1	POLB	RMND1	CMC2
COX4I1	ETFA	ACACA	LYRM2	LIAS
ARF5	SLC25A28	MCUR1	AK4	PIF1
COX7A2	AK3	OXR1	GTPBP3	COX7B2
MRPL41	C15orf48	PTGES2	CBR4	CKMT1A
FIS1	HSD17B8	SELENOO	AIFM1	IMMP1L
NDUFA4	MRPL39	NDUFS1	TOMM40L	GLYCTK
OCIAD2	ARMCX3	MCCC1	TRIT1	MLYCD
HSPD1	CASP3	SLC25A22	PPOX	COQ6
STOML2	DLD	BID	ECI2	MRPS18C
MRPS21	MRPL36	HSDL1	CHCHD6	GLUD2
HADHA	SFXN3	SLC8B1	THG1L	CPT1C
NDUFA8	OXLD1	METTL5	VPS13D	SDHA
PRDX3	RCC1L	SLC25A24	CASP9	GPAT2
PRDX2	GRSF1	FASTKD5	TOP3A	PRELID2
POLDIP2	PHB	POLRMT	NLN	CA5B

ATP5F1E	COX17	C6orf136	TCAIM	PRORP
NDUFB4	FLAD1	GCAT	NDUFAF7	PTPMT1
AURKAIP1	TMEM126B	MRPL50	TRMT5	PTCD2
FTH1	SPATA20	LETM1	HIBCH	CKMT2
GUK1	COQ10B	ACOT9	FECH	PDE2A
UQCRC1	SFXN4	CPOX	ETFDH	CSKMT
MRPS26	CRYZ	PXMP4	TWNK	POLQ
DBI	TFB2M	OXCT1	MIGA2	C5orf63
MRPS18B	ALDH1B1	CHCHD4	APOOL	GLDC
OAT	DTYMK	COMTD1	SLC25A12	FHIT
TIMM23	ECI1	HAGH	MYO19	SEPTIN4
FASN	SDHAF3	MTHFD1L	OXSM	ACOT11
SND1	IDI1	SLC25A44	PNPO	LYRM9
PHB2	PGAM5	LYRM1	C12orf65	PXMP2
MGST1	BAK1	ETFRF1	FASTKD1	NAT8L
SLC25A1	MCU	HDHD5	SLC25A15	ACADL
STARD7	MCCC2	SLC25A36	NSUN4	MICU3
NDUFB3	CHCHD3	SNAP29	PDE12	C3orf33
CYB5R3	FUNDC1	LYPLAL1	OMA1	AGPAT4
IARS2	ALDH3A2	YARS2	PANK2	NDUFV2
TXN2	PLGRKT	CDK5RAP1	COX18	SERHL2
MRPL24	NIF3L1	SUOX	MARS2	FXN
ATP5MC3	HINT3	RHOT1	BBC3	DMGDH
MRPS35	NDUFS3	ACOT2	PDK2	MRPL2
ATP5PB	MRPL27	MRPL30	IDE	OGDHL
NDUFA6	SSBP1	PGS1	NDUFAF1	DNAJC28
GSR	MTIF3	MRPS5	DMPK	CYP11A1
PRELID3B	TMEM205	SFXN1	FAM210A	MTG1
SDHC	MRPS36	MRPS33	MRPS17	ACSS3
FKBP10	PPA2	RMDN1	EPHX2	KMO
KARS1	MRPL52	MAOB	MRPL42	NDUFS7
GLUD1	TRMT10C	NNT	FBXL4	BCL2L10
BNIP3L	TIMM9	MRPS30	NFS1	SMIM8
LAP3	HIGD1A	TRMT2B	SUCLA2	SARS2
SLC25A3	SLC25A38	SDHAF4	SLC25A10	CRLS1
ACLY	MMUT	HTRA2	POLG2	ETFBKMT
AHCYL1	C16orf91	GUF1	PDPR	PET100
ECH1	LACTB2	MRPS31	QRSL1	AFG1L
MMADHC	NDUFS2	CHCHD7	FARS2	MRPL38
TOMM22	MSRB2	ACAT1	MSRB3	NIPSNAP3B
COA3	NDUFB5	COX15	ACAD10	SLC25A21
ATP5IF1	NDUFAF8	ACADSB	PINK1	LETM2
MRPL18	NT5DC2	HSCB	ACCS	AIFM3

ATP5F1A	ARL2	MTARC2	CEP89	C15orf61
UQCRC2	GPT2	ATP5MG	MRPS22	GDAP1
NDUFAB1	CYB5B	MTX1	DHRS4	PET117
MRPL15	MRPL1	MTG2	COQ8B	CLYBL
MRPL3	MRPL43	TDRKH	EHHADH	PTCD1
ALDH18A1	HSPE1	MCRIP2	CROT	NDUFA13
BCL2L1	FAM162A	NDUFV3	CCDC127	ADHFE1
FH	MFN2	LYRM4	PARL	UCP3
VDAC3	FDPS	AIFM2	NME6	ACSM1
PYCR1	RIDA	DNAJC19	COA8	SLC25A53
SPR	PYCR2	MCAT	NDUFAF6	SCO2
ALAS1	NDUFC1	ABCB10	PARS2	MRPL46
C1QBP	MRPL32	FUNDC2	DMAC2L	TOMM5
DCXR	PDHA1	AKAP10	MTX3	ABCA9
OGDH	BOLA1	CASP8	NAGS	ALDH1L1
TIMM10	RARS2	EFHD1	TIMM21	COX20
MTCH2	TBRG4	PREPL	USP30	AMACR
TST	PRXL2A	MALSU1	ME2	MTFP1
SDHB	MTIF2	BCS1L	SLC25A26	FMC1
OCIAD1	NOA1	MRPS9	MIGA1	GCSH
SOD2	NUDT5	MRPL12	ALDH7A1	SLC25A18
TSTD1	ATAD1	MCEE	OPA3	SLC25A34
COX6C	SLC30A9	BAD	MTFMT	ABCD2
PNKD	IVD	PICK1	MTPAP	PRSS35
CAT	ECSIT	MRPL4	SLC25A42	MGARP
UQCRQ	COQ5	NARS2	BLOC1S1	STAR
HADHB	ECHDC1	ACSS1	GLRX2	TSTD3
DAP3	HEBP1	COX11	CBR3	NUDT6
MRPL37	TMEM70	COA7	VWA8	MTHFS
GSTK1	PDK4	THEM5	AGK	COX8C
MRPL54	DECR1	ISCA2	LIG3	TOMM20L
SUCLG2	MRPL21	ABCD1	SLC25A14	NMNAT3
MRPL33	SPTLC2	PTCD3	PDK3	OXCT2
MRPS10	HSD17B4	NDUFB9	THNSL1	DIABLO
NME3	MARCHF5	ATP5PO	TRMU	BCO2
HSPA9	CRAT	NUDT8	MSRA	ACSM5
CHCHD10	DUT	LACTB	DBT	NDUFA7
NDUFS4	BCL2A1	COX4I2	DUS2	MCCD1
COX14	MTERF3	PISD	SLC25A33	PABPC5
ALKBH7	SLC25A13	SUPV3L1	GATM	COX6B2
NME4	SLC25A43	MTHFD2	TRNT1	CYP11B1
COX7B	MICU2	PCCB	TIMM8A	SLC25A41
HIBADH	DCAKD	MPV17L2	TK2	ABCB6

ATP5PD	PRELID1	MRPL48	SLC25A19	TSFM
MAOA	CPT1A	DNAJC11	SPG7	GATD3A
OXA1L	SDHAF1	AFG3L2	SCO1	PRKN
CYP24A1	YRDC	ACO2	UQCC3	HOGA1
TIMM8B	TARS2	DDX28	SIRT5	PDF
SURF1	RFK	PITRM1	PDSS1	CPT1B
FAM210B	DNAJC4	ANTKMT	MRPS28	MRPS24
NDUFV1	MRPS14	TIMM22	BPHL	MUTYH
HTATIP2	SLC25A37	ARMCX1	MOCS1	FABP1
PEX11B	ACADS	UQCR11	CYP27B1	LDHAL6B
MRPS15	CLPP	SLC25A46	MTERF4	MPC1L
LYPLA1	PMAIP1	RPUSD4	FAHD2A	BCKDHA
TIMM13	GPD2	ALDH6A1	GPAM	CA5A
CS	TIMM10B	POLG	COX19	SLC25A32
HINT1	MGST3	MCUB	MRRF	ALAS2
MRPS16	TMEM11	TRUB2	ACP6	COQ8A
PPIF	ACADM	ALDH4A1	MRPS11	ACSM4
GOT2	PCK2	TIMM44	XPNPEP3	NEU4
UQCR10	RMDN3	CCDC51	PPM1K	PRODH2
MICOS13	COA5	PC	SLC25A51	ACSL6
CPS1	NUDT9	LDHB	GFER	VARS2
IMMT	PHYH	SYNJ2BP	PRIMPOL	HINT2
AKR7A2	DLAT	YBEY	PDK1	ACOD1
MRPL47	TACO1	MIEF1	PCCA	ACAD11
MTX2	DHX30	ECHDC2	ETFB	GLS2
MRPL45	ISOC2	SAMM50	LYRM7	COX6A2
IDH3B	MGME1	ACAA1	GSTZ1	AGXT
SHMT2	SLIRP	PIGBOS1	IDH3A	AMT
YME1L1	GLOD4	NDUFC2	MTERF1	SLC25A48
CYP27A1	NTHL1	DGLUCY	OSGEPL1	DNLZ
COA4	HCCS	ACOT13	IMMP2L	OTC
DLST	MRPS23	BCL2L11	CHDH	ADCY10
MRPL49	ABCD3	SDHAF2	SLC25A30	MRM2
ALDH9A1	BCAT2	ADCK5	SLC25A35	AGXT2
MRPL58	DHTKD1	UQCC1	METTL4	PTRH1
CYCS	CISD3	DNAJC15	NOCT	SLC25A47
COASY	DMAC1	MRPL19	HMGCS2	SLC25A52
ACSL1	NUDT19	RECQL4	COX10	MYG1
NDUFAF3	NUDT2	TOP1MT	ARG2	SPHKAP
BAX	COQ9	GFM2	SFXN2	HTD2
NIPSNAP1	ACSF2	SLC25A29	COQ10A	UCP1
SLC25A11	KYAT3	PTRH2	TFB1M	FDX2
NDUFB6	DMAC2	PDSS2	SPHK2	PLSCR3

GLS	RDH14	APOO	AADAT	HAO2
CHCHD1	MPST	DNAJC30	MTERF2	ATP5MF-PTCD1
ABHD11	NDUFB2	DHRS1	MTRF1	SPATA19
ERAL1	COQ4	METTL17	NDUFAF2	SLC25A31
FASTK	ETHE1	DARS2	ATPAF2	ACSM2B
TIMMDC1	SLC25A20	CHPT1	BDH1	GLYAT
VDAC2	ACOT7	RTL10	OXNAD1	CYP11B2
TOMM34	PDP1	MRM1	DHODH	PDHA2
MRPL17	MFF	COQ2	GTPBP10	ACSM2A
ATP5PF	UQCRB	AUH	ABAT	MRPL53
IDH3G	FDX1	EXD2	NT5DC3	FTMT
GADD45GIP1	PMPCB	ARMCX6	PDP2	PYURF
SMDT1	DELE1	RNASEH1	CLPB	CMC4
MRPL20	MAVS	CRY1	DNA2	TOMM6

Table S3. Summary of hypoxia score and mitochondrial score in TCGA-LUAD patients

	hypoxia	mitochondrial
TCGA-35-5375-01A-01R-1628-07	3.529081878	3.630882555
TCGA-55-A4DF-01A-11R-A24H-07	3.537711699	3.45522796
TCGA-95-8039-01A-11R-2241-07	3.433721946	3.476135161
TCGA-MP-A4T4-01A-11R-A262-07	3.504607283	3.457777798
TCGA-62-A471-01A-12R-A24H-07	3.550609009	3.590620525
TCGA-L9-A5IP-01A-21R-A39D-07	3.580881052	3.522274958
TCGA-50-5936-01A-11R-1628-07	3.591794064	3.519890191
TCGA-49-AARR-01A-11R-A41B-07	3.510131488	3.383876511
TCGA-78-8662-01A-11R-2403-07	3.367944658	3.576235683
TCGA-05-4430-01A-02R-1206-07	3.528017407	3.5009118
TCGA-44-7659-01A-11R-2066-07	3.347968675	3.508578222
TCGA-44-6146-01A-11R-A278-07	3.363036145	3.403655056
TCGA-L9-A50W-01A-12R-A39D-07	3.470431076	3.453906961
TCGA-05-4424-01A-22R-1858-07	3.555709458	3.470529541
TCGA-62-A46P-01A-11R-A24H-07	3.443543642	3.581883782
TCGA-38-4627-01A-01R-1206-07	3.651028182	3.494150942
TCGA-86-8073-01A-11R-2241-07	3.438225343	3.452453149
TCGA-55-6984-01A-11R-1949-07	3.555043782	3.469143991
TCGA-69-7764-01A-11R-2170-07	3.375636225	3.466692063
TCGA-86-A4P7-01A-11R-A24X-07	3.461830839	3.437283998
TCGA-55-8620-01A-11R-2403-07	3.414332884	3.509422068
TCGA-97-8177-01A-11R-2287-07	3.591813085	3.557904636
TCGA-50-6595-01A-12R-1858-07	3.628785774	3.462145825

TCGA-55-A492-01A-11R-A24H-07	3.361609826	3.630901897
TCGA-78-7633-01A-11R-2066-07	3.443005878	3.584991187
TCGA-97-A4LX-01A-11R-A24X-07	3.477214186	3.445333471
TCGA-78-7220-01A-11R-2039-07	3.515513904	3.575899261
TCGA-53-7624-01A-11R-2066-07	3.557609838	3.500074658
TCGA-49-AARN-01A-21R-A41B-07	3.509873587	3.494484865
TCGA-62-A46R-01A-11R-A24H-07	3.334640148	3.493147713
TCGA-78-7156-01A-11R-2039-07	3.293580246	3.576150276
TCGA-50-8457-01A-11R-2326-07	3.479152025	3.446619323
TCGA-91-8496-01A-11R-2403-07	3.445428368	3.599852492
TCGA-55-8092-01A-11R-2241-07	3.404926403	3.471300979
TCGA-NJ-A4YQ-01A-11R-A262-07	3.422688332	3.473140037
TCGA-50-5072-01A-21R-1858-07	3.610911966	3.532384686
TCGA-91-6831-01A-11R-1858-07	3.565376752	3.513276774
TCGA-91-A4BD-01A-11R-A24H-07	3.387154776	3.587407591
TCGA-69-8255-01A-11R-2287-07	3.395919175	3.638754177
TCGA-69-8253-01A-11R-2287-07	3.551165797	3.554325474
TCGA-86-A4D0-01A-11R-A24H-07	3.440443369	3.60496114
TCGA-78-8640-01A-11R-2403-07	3.443600545	3.533246745
TCGA-97-8174-01A-11R-2287-07	3.452426735	3.467918637
TCGA-55-6987-01A-11R-1949-07	3.545429298	3.495796286
TCGA-49-6744-01A-11R-1858-07	3.552482445	3.476167641
TCGA-55-8096-01A-11R-2241-07	3.573696537	3.449277323
TCGA-J2-8192-01A-11R-2241-07	3.593804746	3.386572913
TCGA-44-7671-01A-11R-2066-07	3.521232347	3.557302883
TCGA-44-2662-01B-02R-A277-07	3.227284471	2.758172862
TCGA-93-A4JN-01A-11R-A24X-07	3.459480235	3.394874009
TCGA-49-AAR9-01A-21R-A41B-07	3.537614876	3.522074036
TCGA-86-A4P8-01A-11R-A24X-07	3.44040234	3.401891467
TCGA-78-7158-01A-11R-2039-07	3.262160526	3.536749599
TCGA-44-4112-01A-01R-1107-07	3.601636039	3.53731912
TCGA-71-6725-01A-11R-1858-07	3.295950292	3.509082425
TCGA-44-2656-01A-02R-A278-07	3.334992664	3.362636231
TCGA-05-5715-01A-01R-1628-07	3.484380271	3.531101135
TCGA-64-5815-01A-01R-1628-07	3.715508755	3.440100391
TCGA-55-7816-01A-11R-2170-07	3.444351199	3.447514628
TCGA-95-7948-01A-11R-2187-07	3.336663771	3.55665465
TCGA-05-4432-01A-01R-1206-07	3.621056295	3.530672
TCGA-99-8033-01A-11R-2241-07	3.556125034	3.500077431
TCGA-78-7143-01A-11R-2039-07	3.403233343	3.461368208
TCGA-73-4668-01A-01R-1206-07	3.589423364	3.489973889
TCGA-38-4625-01A-01R-1206-07	3.445176194	3.564719401
TCGA-91-8499-01A-11R-2403-07	3.466631004	3.585045506

TCGA-86-6851-01A-11R-1949-07	3.449570256	3.406524039
TCGA-44-6779-01A-11R-1858-07	3.492329862	3.423687582
TCGA-78-7537-01A-11R-2066-07	3.409518677	3.539930994
TCGA-05-4434-01A-01R-1206-07	3.636082564	3.496202679
TCGA-67-3773-01A-01R-0946-07	3.433058433	3.55105667
TCGA-55-6983-01A-11R-1949-07	3.494618672	3.460352516
TCGA-05-5425-01A-02R-1628-07	3.539646585	3.595416132
TCGA-97-A4M2-01A-12R-A24X-07	3.369252565	3.480584438
TCGA-50-5941-01A-11R-1755-07	3.496038108	3.494537324
TCGA-78-7539-01A-11R-2066-07	3.362734502	3.527078135
TCGA-67-3771-01A-01R-0946-07	3.5761663	3.471395597
TCGA-55-8299-01A-11R-2287-07	3.559574986	3.481097798
TCGA-05-5429-01A-01R-1628-07	3.583702098	3.59585641
TCGA-93-A4JO-01A-21R-A24X-07	3.407890439	3.431685924
TCGA-44-A47A-01A-21R-A24H-07	3.439921353	3.500158532
TCGA-44-2662-01A-01R-0946-07	3.647329251	3.466721699
TCGA-86-A4JF-01A-11R-A24X-07	3.514435178	3.526541234
TCGA-05-4402-01A-01R-1206-07	3.542146443	3.501770956
TCGA-97-8179-01A-11R-2287-07	3.372887604	3.554990413
TCGA-55-7914-01A-11R-2170-07	3.40298066	3.497420052
TCGA-49-4490-01A-21R-1858-07	3.533612127	3.519554139
TCGA-55-8090-01A-11R-2241-07	3.572769224	3.439588267
TCGA-MP-A4TC-01A-11R-A24X-07	3.610634857	3.469330547
TCGA-64-1681-01A-11R-2066-07	3.513007495	3.487263223
TCGA-99-AA5R-01A-11R-A39D-07	3.519635072	3.421736881
TCGA-49-4486-01A-01R-1206-07	3.367034738	3.696271173
TCGA-91-6847-01A-11R-1949-07	3.390008609	3.520753727
TCGA-05-4249-01A-01R-1107-07	3.411881054	3.454727839
TCGA-91-A4BC-01A-11R-A24H-07	3.46390693	3.451536139
TCGA-55-7570-01A-11R-2039-07	3.346516406	3.579309087
TCGA-50-5049-01A-01R-1628-07	3.547631319	3.517967985
TCGA-86-8672-01A-21R-2403-07	3.540598962	3.534738407
TCGA-50-5066-02A-11R-2090-07	3.551623429	3.494226173
TCGA-55-7727-01A-11R-2170-07	3.366157724	3.444342417
TCGA-86-8674-01A-21R-2403-07	3.444457721	3.61940745
TCGA-53-A4EZ-01A-12R-A24X-07	3.364612663	3.599360665
TCGA-69-7761-01A-11R-2170-07	3.504825765	3.382411803
TCGA-50-5942-01A-21R-1755-07	3.416053648	3.461087329
TCGA-69-A59K-01A-11R-A262-07	3.464714924	3.407187577
TCGA-44-2666-01A-01R-A278-07	3.317949305	3.444259897
TCGA-50-5944-01A-11R-1755-07	3.429512928	3.486275705
TCGA-50-6592-01A-11R-1755-07	3.585389438	3.460307736
TCGA-62-8395-01A-11R-2326-07	3.502383985	3.529395303

TCGA-55-6975-01A-11R-1949-07	3.559639002	3.507621575
TCGA-73-4662-01A-01R-1206-07	3.415912008	3.481205251
TCGA-49-6767-01A-11R-1858-07	3.449947259	3.527240851
TCGA-44-A47G-01A-21R-A24H-07	3.59203782	3.451107463
TCGA-NJ-A4YG-01A-22R-A262-07	3.409986878	3.532912591
TCGA-55-8507-01A-11R-2403-07	3.501244262	3.517584437
TCGA-05-4433-01A-22R-1858-07	3.474863934	3.43840442
TCGA-55-A4DG-01A-11R-A24H-07	3.343981944	3.453784174
TCGA-49-4494-01A-01R-1206-07	3.555853609	3.591376015
TCGA-NJ-A7XG-01A-12R-A39D-07	3.359559307	3.525391276
TCGA-44-6147-01A-11R-A278-07	3.393714158	3.299975524
TCGA-49-AARO-01A-12R-A41B-07	3.551347537	3.439449916
TCGA-44-6774-01A-21R-1858-07	3.605455428	3.441783898
TCGA-49-AAR4-01A-12R-A41B-07	3.473153994	3.447288072
TCGA-91-6848-01A-11R-1949-07	3.523966172	3.423120099
TCGA-50-8460-01A-11R-2326-07	3.443510283	3.516825338
TCGA-44-2668-01B-02R-A277-07	3.315583747	2.857739815
TCGA-55-8091-01A-11R-2241-07	3.59250165	3.443514623
TCGA-50-6673-01A-11R-1949-07	3.568577134	3.472464106
TCGA-49-AARE-01A-11R-A41B-07	3.462767187	3.501501823
TCGA-55-6985-01A-11R-1949-07	3.541317204	3.413381537
TCGA-55-6642-01A-11R-1858-07	3.627138354	3.428636232
TCGA-50-6591-01A-11R-1755-07	3.488172976	3.47431516
TCGA-78-7166-01A-12R-2066-07	3.580585292	3.597705507
TCGA-05-4427-01A-21R-1858-07	3.479741274	3.431851293
TCGA-49-4487-01A-21R-1858-07	3.575168501	3.499815168
TCGA-73-4676-01A-01R-1755-07	3.571529388	3.600270574
TCGA-49-4507-01A-01R-1206-07	3.487945417	3.600006904
TCGA-78-7148-01A-11R-2039-07	3.567088708	3.525146409
TCGA-55-8514-01A-11R-2403-07	3.40012645	3.508811228
TCGA-93-A4JQ-01A-11R-A24X-07	3.507034356	3.410622393
TCGA-55-7576-01A-11R-2066-07	3.449279065	3.477976192
TCGA-55-6969-01A-11R-1949-07	3.583950736	3.494547539
TCGA-93-7347-01A-11R-2187-07	3.458149028	3.462123775
TCGA-69-7978-01A-11R-2187-07	3.616509567	3.406749857
TCGA-NJ-A4YF-01A-12R-A262-07	3.519321205	3.630790651
TCGA-91-6830-01A-11R-1949-07	3.620009755	3.446468498
TCGA-44-7672-01A-11R-2066-07	3.565502841	3.44990864
TCGA-78-7540-01A-11R-2066-07	3.485653517	3.459574652
TCGA-55-7227-01A-11R-2039-07	3.476203905	3.44967855
TCGA-MP-A4T6-01A-32R-A262-07	3.22065446	3.447670778
TCGA-99-8028-01A-11R-2241-07	3.610200308	3.47348008
TCGA-64-1677-01A-01R-0946-07	3.443922026	3.598090407

TCGA-05-4403-01A-01R-1206-07	3.573195944	3.511939724
TCGA-44-6148-01A-11R-1755-07	3.465610209	3.420655208
TCGA-55-6980-01A-11R-1949-07	3.634179869	3.464088961
TCGA-55-8511-01A-11R-2403-07	3.472422175	3.464770143
TCGA-86-8278-01A-11R-2287-07	3.500178165	3.445445839
TCGA-78-7167-01A-11R-2066-07	3.328309107	3.545401085
TCGA-55-8615-01A-11R-2403-07	3.501826775	3.527467808
TCGA-55-A494-01A-11R-A24X-07	3.315544548	3.475113645
TCGA-78-7147-01A-11R-2039-07	3.401828535	3.545823413
TCGA-97-A4M0-01A-11R-A24X-07	3.362388596	3.450041732
TCGA-55-7573-01A-11R-2039-07	3.411200965	3.406238971
TCGA-86-7714-01A-12R-2170-07	3.410485421	3.467688285
TCGA-64-1679-01A-21R-2066-07	3.657445652	3.422347262
TCGA-64-1680-01A-02R-0946-07	3.394522426	3.571449289
TCGA-86-8669-01A-11R-2403-07	3.526464422	3.522419214
TCGA-78-7145-01A-11R-2039-07	3.568904102	3.516399033
TCGA-86-8075-01A-11R-2241-07	3.627125034	3.421863788
TCGA-99-8025-01A-11R-2241-07	3.479835767	3.500202262
TCGA-L9-A743-01A-43R-A39D-07	3.575943857	3.440678949
TCGA-86-8054-01A-11R-2241-07	3.560293628	3.552535812
TCGA-55-8094-01A-11R-2241-07	3.471667929	3.56193482
TCGA-62-A470-01A-11R-A24H-07	3.464724014	3.572208795
TCGA-44-2666-01A-01R-0946-07	3.43709505	3.530054327
TCGA-86-A456-01A-11R-A24H-07	3.458990285	3.503872243
TCGA-MP-A4SW-01A-21R-A24X-07	3.462578575	3.478623306
TCGA-44-3919-01A-02R-1107-07	3.513544502	3.46298685
TCGA-69-7973-01A-11R-2187-07	3.537507175	3.511143291
TCGA-86-8671-01A-11R-2403-07	3.487116629	3.418923956
TCGA-67-3772-01A-01R-0946-07	3.527768161	3.526665236
TCGA-35-4122-01A-01R-1107-07	3.563901265	3.601772904
TCGA-55-8513-01A-11R-2403-07	3.466064501	3.40214823
TCGA-35-4123-01A-01R-1107-07	3.552629049	3.567043417
TCGA-49-6761-01A-31R-1949-07	3.560390078	3.47871468
TCGA-55-6982-01A-11R-1949-07	3.585776786	3.465538516
TCGA-44-A4SU-01A-11R-A24X-07	3.459349908	3.464120521
TCGA-86-8279-01A-11R-2287-07	3.489666448	3.490283184
TCGA-05-4396-01A-21R-1858-07	3.460259026	3.491190991
TCGA-86-8358-01A-11R-2326-07	3.520684558	3.482415764
TCGA-55-7815-01A-11R-2170-07	3.502725855	3.426282113
TCGA-78-7162-01A-21R-2066-07	3.474369526	3.515939647
TCGA-MN-A4N1-01A-11R-A24X-07	3.43506865	3.534785321
TCGA-44-2668-01A-01R-A278-07	3.576764314	3.338489751
TCGA-44-6777-01A-11R-1858-07	3.669750073	3.435408015

TCGA-55-8301-01A-11R-2287-07	3.51704383	3.509733572
TCGA-05-4422-01A-01R-1206-07	3.365189611	3.58600857
TCGA-49-4506-01A-01R-1206-07	3.51303188	3.645723857
TCGA-05-4417-01A-22R-1858-07	3.581291451	3.483155054
TCGA-62-A46O-01A-11R-A24H-07	3.408208301	3.542107556
TCGA-97-8552-01A-11R-2403-07	3.41055158	3.480938287
TCGA-MP-A5C7-01A-11R-A262-07	3.290002963	3.519092736
TCGA-86-8074-01A-11R-2241-07	3.569730287	3.447593009
TCGA-97-7937-01A-11R-2170-07	3.367331767	3.492341345
TCGA-97-8175-01A-11R-2287-07	3.596889368	3.503226013
TCGA-97-A4M7-01A-11R-A24X-07	3.49616098	3.410030883
TCGA-05-4420-01A-01R-1206-07	3.423841439	3.573582818
TCGA-91-8497-01A-11R-2403-07	3.488112298	3.488211138
TCGA-38-4632-01A-01R-1755-07	3.515746139	3.579310785
TCGA-69-7979-01A-11R-2187-07	3.462395096	3.509259564
TCGA-78-7163-01A-12R-2066-07	3.454607945	3.583548122
TCGA-86-7701-01A-11R-2170-07	3.558877746	3.414455076
TCGA-67-3770-01A-01R-0946-07	3.461577603	3.599223182
TCGA-38-4629-01A-02R-1206-07	3.660161197	3.555348234
TCGA-MP-A4TA-01A-21R-A24X-07	3.425260441	3.560619517
TCGA-50-5931-01A-11R-1755-07	3.590805348	3.535380948
TCGA-55-7994-01A-11R-2187-07	3.384540069	3.4386183
TCGA-55-7724-01A-11R-2170-07	3.525277412	3.405666159
TCGA-55-7725-01A-11R-2170-07	3.337437538	3.465702708
TCGA-MP-A4SY-01A-21R-A24X-07	3.551101314	3.498211199
TCGA-97-7546-01A-11R-2039-07	3.451074177	3.40815751
TCGA-78-7154-01A-11R-2039-07	3.52025413	3.555670029
TCGA-73-4659-01A-01R-1206-07	3.597493257	3.541589923
TCGA-69-7980-01A-11R-2187-07	3.472335315	3.441778354
TCGA-73-7498-01A-12R-2187-07	3.360770985	3.51188411
TCGA-86-8585-01A-11R-2403-07	3.566282175	3.531341144
TCGA-78-7542-01A-21R-2066-07	3.511002609	3.486388061
TCGA-05-4250-01A-01R-1107-07	3.62253362	3.532656018
TCGA-44-6776-01A-11R-1858-07	3.409766347	3.575424113
TCGA-38-4630-01A-01R-1206-07	3.441453941	3.543138029
TCGA-05-4418-01A-01R-1206-07	3.590717438	3.613788282
TCGA-55-8085-01A-11R-2241-07	3.485231617	3.567150607
TCGA-55-7995-01A-11R-2187-07	3.529039347	3.466776352
TCGA-55-7284-01B-11R-2241-07	3.545605255	3.446365812
TCGA-55-6979-01A-11R-1949-07	3.494449827	3.460399377
TCGA-62-A472-01A-11R-A24H-07	3.43287095	3.508725933
TCGA-55-8204-01A-11R-2241-07	3.516772386	3.430138266
TCGA-62-8398-01A-11R-2326-07	3.600634381	3.547248349

TCGA-MP-A4TI-01A-21R-A24X-07	3.56549841	3.443350673
TCGA-44-2656-01A-02R-0946-07	3.4756309	3.477304427
TCGA-05-4390-01A-02R-1755-07	3.573687252	3.540980153
TCGA-44-8120-01A-11R-2241-07	3.485415979	3.487950713
TCGA-O1-A52J-01A-11R-A262-07	3.455764595	3.488437163
TCGA-67-6217-01A-11R-1755-07	3.401461672	3.465419685
TCGA-44-6146-01A-11R-1755-07	3.438351696	3.473807274
TCGA-MP-A4TK-01A-11R-A24X-07	3.651101956	3.433666776
TCGA-MN-A4N4-01A-12R-A24X-07	3.572427394	3.42477254
TCGA-73-4666-01A-01R-1206-07	3.471584276	3.54785773
TCGA-L4-A4E6-01A-11R-A24H-07	3.464256753	3.456298499
TCGA-55-8207-01A-11R-2241-07	3.554133972	3.44120631
TCGA-38-A44F-01A-11R-A24H-07	3.540207684	3.435240298
TCGA-78-7536-01A-11R-2066-07	3.419679948	3.519623578
TCGA-05-4389-01A-01R-1206-07	3.424016274	3.583643959
TCGA-55-7281-01A-11R-2039-07	3.538079637	3.463769066
TCGA-97-7547-01A-11R-2039-07	3.458254	3.445042588
TCGA-62-8394-01A-11R-2326-07	3.538278648	3.526589721
TCGA-86-8668-01A-11R-2403-07	3.472665335	3.421176114
TCGA-50-5939-01A-11R-1628-07	3.661461836	3.477404524
TCGA-38-7271-01A-11R-2039-07	3.525235184	3.432612529
TCGA-49-AARQ-01A-11R-A41B-07	3.330526117	3.550194057
TCGA-49-AAR3-01A-11R-A41B-07	3.430017532	3.501114091
TCGA-44-A479-01A-31R-A24H-07	3.454488107	3.435466788
TCGA-49-4514-01A-21R-1858-07	3.432420408	3.61890215
TCGA-62-A46S-01A-11R-A24H-07	3.4746088	3.502229922
TCGA-55-A490-01A-11R-A466-07	3.538038707	3.433689636
TCGA-55-8097-01A-11R-2241-07	3.287091777	3.44348288
TCGA-55-8208-01A-11R-2241-07	3.522341218	3.426741553
TCGA-69-7974-01A-11R-2187-07	3.541507731	3.448744716
TCGA-55-6968-01A-11R-1949-07	3.479796967	3.490823545
TCGA-44-3917-01B-02R-A277-07	2.762695727	2.954992503
TCGA-05-4415-01A-22R-1858-07	3.527490414	3.582382029
TCGA-44-6145-01A-11R-1755-07	3.56919255	3.448986295
TCGA-44-7667-01A-31R-2066-07	3.363398406	3.572342581
TCGA-97-7553-01A-21R-2039-07	3.478338712	3.406083947
TCGA-78-7150-01A-21R-2039-07	3.633454789	3.521022087
TCGA-69-7763-01A-11R-2170-07	3.540578255	3.473434053
TCGA-95-7947-01A-11R-2187-07	3.409695741	3.51627841
TCGA-55-8089-01A-11R-2241-07	3.458719345	3.394641444
TCGA-86-7711-01A-11R-2066-07	3.525942915	3.502927114
TCGA-55-8621-01A-11R-2403-07	3.544438582	3.426577542
TCGA-49-4501-01A-01R-1206-07	3.517966848	3.563267154

TCGA-L9-A8F4-01A-11R-A39D-07	3.473405672	3.471798383
TCGA-86-7953-01A-11R-2187-07	3.573244364	3.516574497
TCGA-38-4628-01A-01R-1206-07	3.395328064	3.603144671
TCGA-55-A48Y-01A-11R-A24H-07	3.500836483	3.52801589
TCGA-MP-A4T9-01A-11R-A24X-07	3.630125411	3.47041533
TCGA-91-6835-01A-11R-1858-07	3.456656723	3.39547848
TCGA-50-6590-01A-12R-1858-07	3.562438955	3.482867079
TCGA-95-7039-01A-11R-1949-07	3.507630866	3.515865892
TCGA-55-8206-01A-11R-2241-07	3.380797528	3.45545922
TCGA-44-7661-01A-11R-2066-07	3.675047053	3.449124055
TCGA-J2-A4AD-01A-11R-A24H-07	3.473740763	3.493843983
TCGA-55-8616-01A-11R-2403-07	3.45514837	3.52008698
TCGA-50-5051-01A-21R-1858-07	3.51858929	3.544983838
TCGA-97-A4M3-01A-11R-A24X-07	3.522307819	3.549872811
TCGA-97-7552-01A-11R-2039-07	3.481376248	3.376675216
TCGA-44-2657-01A-01R-1107-07	3.471055689	3.476604105
TCGA-97-8172-01A-11R-2287-07	3.387573109	3.432476372
TCGA-44-2662-01A-01R-A278-07	3.57516709	3.387231966
TCGA-91-6840-01A-11R-1949-07	3.37888904	3.462664265
TCGA-55-7907-01A-11R-2170-07	3.436326017	3.452444094
TCGA-49-4510-01A-01R-1206-07	3.534222824	3.573752134
TCGA-MP-A4SV-01A-11R-A24X-07	3.456949167	3.486708302
TCGA-55-7726-01A-11R-2170-07	3.557106829	3.415870329
TCGA-97-A4M6-01A-11R-A24X-07	3.488950637	3.474812266
TCGA-86-7955-01A-11R-2187-07	3.38592152	3.603785428
TCGA-44-4112-01B-06R-A277-07	3.140429201	2.775462945
TCGA-L9-A443-01A-12R-A24H-07	3.480727137	3.481531267
TCGA-53-7813-01A-11R-2170-07	3.386029841	3.50207869
TCGA-55-A491-01A-11R-A24H-07	3.557282836	3.483002339
TCGA-86-8056-01A-11R-2241-07	3.412940717	3.434259767
TCGA-78-8655-01A-11R-2403-07	3.399140733	3.499104009
TCGA-44-6147-01A-11R-1755-07	3.536133133	3.437976491
TCGA-44-6147-01B-06R-A277-07	3.053839798	2.715508755
TCGA-L9-A444-01A-21R-A24H-07	3.448249438	3.444950013
TCGA-86-8055-01A-11R-2241-07	3.662054138	3.401361865
TCGA-55-6970-01A-11R-1949-07	3.569871481	3.552505012
TCGA-49-6745-01A-11R-1858-07	3.567322064	3.469551804
TCGA-05-4405-01A-21R-1858-07	3.46281945	3.440381528
TCGA-MP-A4TH-01A-31R-A262-07	3.371919507	3.423080225
TCGA-99-7458-01A-11R-2039-07	3.501442272	3.430719952
TCGA-55-8508-01A-11R-2403-07	3.587925863	3.468258526
TCGA-55-1594-01A-01R-0946-07	3.455369938	3.526991184
TCGA-44-7669-01A-21R-2066-07	3.518361777	3.502626596

TCGA-49-6742-01A-11R-1858-07	3.539519209	3.545720086
TCGA-55-7913-01B-11R-2241-07	3.422695604	3.567685208
TCGA-86-6562-01A-11R-1755-07	3.571694486	3.465298016
TCGA-95-7944-01A-11R-2187-07	3.444107994	3.540223441
TCGA-05-4398-01A-01R-1206-07	3.610479565	3.493897839
TCGA-78-7161-01A-11R-2039-07	3.459895948	3.461619062
TCGA-55-7728-01A-11R-2187-07	3.449414472	3.433691699
TCGA-55-8087-01A-11R-2241-07	3.26654668	3.465962729
TCGA-91-7771-01A-11R-2170-07	3.5544545	3.458511096
TCGA-67-6216-01A-11R-1755-07	3.493190624	3.507085492
TCGA-95-A4VP-01A-21R-A262-07	3.551688418	3.497271378
TCGA-73-4675-01A-01R-1206-07	3.529132503	3.520679389
TCGA-64-1676-01A-01R-0946-07	3.495943805	3.614560091
TCGA-55-6981-01A-11R-1949-07	3.500863478	3.49944243
TCGA-L9-A7SV-01A-11R-A39D-07	3.253568664	3.538666321
TCGA-78-7160-01A-11R-2039-07	3.552616456	3.489612413
TCGA-55-1595-01A-01R-0946-07	3.412111808	3.481123013
TCGA-78-8648-01A-11R-2403-07	3.646762356	3.420136112
TCGA-44-3918-01B-02R-A277-07	3.039158539	2.72335338
TCGA-55-A57B-01A-12R-A39D-07	3.46939101	3.437464339
TCGA-78-7535-01A-11R-2066-07	3.442145189	3.489697054
TCGA-55-6986-01A-11R-1949-07	3.509236655	3.536809431
TCGA-91-6849-01A-11R-1949-07	3.510305061	3.52968985
TCGA-55-7903-01A-11R-2170-07	3.439348678	3.485471882
TCGA-55-A493-01A-11R-A24H-07	3.596314579	3.426745521
TCGA-86-8280-01A-11R-2287-07	3.502351621	3.476593111
TCGA-91-6828-01A-11R-1858-07	3.512517253	3.429301273
TCGA-55-A48X-01A-11R-A24H-07	3.416767404	3.416605913
TCGA-44-3917-01A-01R-A278-07	3.18834076	3.47566087
TCGA-05-5423-01A-01R-1628-07	3.41394869	3.606430447
TCGA-05-5420-01A-01R-1628-07	3.482296348	3.633511363
TCGA-62-A46Y-01A-11R-A24H-07	3.455806541	3.522944035
TCGA-05-4384-01A-01R-1755-07	3.474371283	3.523299676
TCGA-97-A4M1-01A-11R-A24X-07	3.338474553	3.518999328
TCGA-95-7567-01A-11R-2066-07	3.423030398	3.5106284
TCGA-55-7910-01A-11R-2170-07	3.513742801	3.477561263
TCGA-55-8619-01A-11R-2403-07	3.547031207	3.414474682
TCGA-55-8302-01A-11R-2326-07	3.536589572	3.497762761
TCGA-MN-A4N5-01A-11R-A24X-07	3.459273889	3.469912183
TCGA-95-A4VK-01A-11R-A262-07	3.441657733	3.488590905
TCGA-95-8494-01A-11R-2326-07	3.564592828	3.529473515
TCGA-64-5779-01A-01R-1628-07	3.536550766	3.435653357
TCGA-49-4505-01A-01R-1206-07	3.539057844	3.508080573

TCGA-44-6775-01C-02R-A277-07	3.008536625	2.768671124
TCGA-55-1592-01A-01R-0946-07	3.470900382	3.499563931
TCGA-05-4397-01A-01R-1206-07	3.513823284	3.623338814
TCGA-44-2665-01A-01R-0946-07	3.589602973	3.458677013
TCGA-55-A48Z-01A-12R-A24X-07	3.561349582	3.451084417
TCGA-86-8359-01A-11R-2326-07	3.572140327	3.485055053
TCGA-97-8547-01A-11R-2403-07	3.521478747	3.488930163
TCGA-44-3396-01A-01R-1206-07	3.558310817	3.483154668
TCGA-44-6775-01A-11R-A278-07	3.491636375	3.336434781
TCGA-62-8397-01A-11R-2326-07	3.466219325	3.54696899
TCGA-J2-A4AG-01A-11R-A24H-07	3.52557648	3.454484561
TCGA-44-6775-01A-11R-1858-07	3.548824655	3.432481738
TCGA-93-7348-01A-21R-2039-07	3.542484116	3.444626856
TCGA-64-5775-01A-01R-1628-07	3.527715511	3.504175493
TCGA-78-7153-01A-11R-2039-07	3.429086878	3.616564626
TCGA-78-7149-01A-11R-2039-07	3.439746585	3.597434009
TCGA-MP-A4T8-01A-11R-A24X-07	3.551700032	3.552387477
TCGA-44-2661-01A-01R-1107-07	3.421677437	3.533045439
TCGA-44-2656-01B-06R-A277-07	3.024140298	2.844120696
TCGA-44-6778-01A-11R-1858-07	3.524586967	3.417060152
TCGA-44-7670-01A-11R-2066-07	3.524080341	3.483264795
TCGA-55-8205-01A-11R-2241-07	3.476838311	3.492677217
TCGA-55-8512-01A-11R-2403-07	3.450592807	3.451909241
TCGA-55-8203-01A-11R-2241-07	3.434460719	3.471250468
TCGA-78-8660-01A-11R-2403-07	3.465782893	3.503632308
TCGA-44-A47B-01A-11R-A24H-07	3.476336983	3.446732286
TCGA-50-5946-01A-11R-1755-07	3.382260835	3.445870558
TCGA-38-4631-01A-01R-1755-07	3.494060452	3.620336345
TCGA-64-5778-01A-01R-1628-07	3.388149016	3.576974686
TCGA-86-7954-01A-11R-2187-07	3.395524719	3.45530231
TCGA-50-5066-01A-01R-1628-07	3.577221566	3.60683793
TCGA-55-6543-01A-11R-1755-07	3.542815254	3.499329308
TCGA-73-7499-01A-11R-2187-07	3.39248571	3.58583635
TCGA-44-3918-01A-01R-1107-07	3.519007398	3.49476095
TCGA-62-8399-01A-21R-2326-07	3.504203508	3.453826128
TCGA-49-AAR0-01A-21R-A39D-07	3.48363217	3.542557752
TCGA-55-7283-01A-11R-2039-07	3.369577885	3.50985734
TCGA-50-6593-01A-11R-1755-07	3.531708116	3.50188016
TCGA-50-8459-01A-11R-2326-07	3.62850819	3.391382073
TCGA-44-3918-01A-01R-A278-07	3.292919507	3.26877209
TCGA-67-3774-01A-01R-0946-07	3.551021624	3.488177914
TCGA-49-AAQV-01A-11R-A39D-07	3.515669521	3.567956006
TCGA-95-7562-01A-11R-2241-07	3.479866082	3.506615217

TCGA-69-7760-01A-11R-2170-07	3.453132875	3.494843317
TCGA-71-8520-01A-11R-2403-07	3.545290634	3.448256644
TCGA-55-8614-01A-11R-2403-07	3.591340188	3.453662019
TCGA-55-7574-01A-11R-2039-07	3.566887002	3.407462392
TCGA-50-5935-01A-11R-1755-07	3.518121579	3.49312787
TCGA-95-7043-01A-11R-1949-07	3.33414429	3.562789234
TCGA-97-7941-01A-11R-2187-07	3.533088644	3.492616213
TCGA-44-7660-01A-11R-2066-07	3.435254408	3.497395091
TCGA-MP-A4T7-01A-11R-A24X-07	3.61657872	3.590228722
TCGA-55-6971-01A-11R-1949-07	3.586659084	3.393393029
TCGA-78-7155-01A-11R-2039-07	3.315142672	3.489040722
TCGA-MP-A4TE-01A-22R-A466-07	3.490505235	3.578586561
TCGA-95-A4VN-01A-11R-A262-07	3.522797906	3.459409583
TCGA-93-8067-01A-11R-2287-07	3.556901169	3.499972702
TCGA-J2-8194-01A-11R-2241-07	3.541511692	3.509762554
TCGA-55-8510-01A-11R-2403-07	3.491660039	3.481431317
TCGA-44-5643-01A-01R-1628-07	3.442366452	3.553787998
TCGA-86-7713-01A-11R-2066-07	3.445827285	3.512196181
TCGA-NJ-A55R-01A-11R-A262-07	3.516098648	3.521851828
TCGA-50-5044-01A-21R-1858-07	3.554883921	3.516443263
TCGA-64-5774-01A-01R-1628-07	3.588891805	3.531594037
TCGA-55-6712-01A-11R-1858-07	3.535088904	3.428977287
TCGA-44-2659-01A-01R-0946-07	3.394358381	3.444842362
TCGA-4B-A93V-01A-11R-A39D-07	3.542127205	3.584857745
TCGA-86-8076-01A-31R-2241-07	3.52046433	3.508735708
TCGA-83-5908-01A-21R-2287-07	3.471362114	3.439228556
TCGA-44-A4SS-01A-11R-A24X-07	3.513116563	3.466490635
TCGA-L4-A4E5-01A-11R-A24X-07	3.379554911	3.485495382
TCGA-97-8171-01A-11R-2287-07	3.242227022	3.6318014
TCGA-93-A4JP-01A-11R-A24X-07	3.484912773	3.448383642
TCGA-S2-AA1A-01A-12R-A39D-07	3.480356793	3.413170866
TCGA-55-6978-01A-11R-1949-07	3.588404485	3.429646491
TCGA-78-7146-01A-11R-2039-07	3.566480831	3.500180266
TCGA-MP-A4TD-01A-32R-A262-07	3.56184813	3.524522801
TCGA-50-5930-01A-11R-1755-07	3.630900254	3.493532728
TCGA-64-5781-01A-01R-1628-07	3.57477483	3.58314033
TCGA-78-7159-01A-11R-2039-07	3.514512761	3.470464307
TCGA-50-7109-01A-11R-2039-07	3.431930584	3.515606642
TCGA-05-5428-01A-01R-1628-07	3.498145892	3.661932296
TCGA-49-4512-01A-21R-1858-07	3.457757871	3.520354256
TCGA-44-6146-01B-04R-A277-07	3.011504125	2.77391578
TCGA-NJ-A4YP-01A-11R-A262-07	3.515123531	3.529806521
TCGA-49-AAR2-01A-11R-A39D-07	3.306636622	3.548622591

TCGA-44-8119-01A-11R-2241-07	3.684688432	3.445429198
TCGA-44-8117-01A-11R-2241-07	3.465012639	3.485226172
TCGA-05-4425-01A-01R-1755-07	3.529915518	3.517607448
TCGA-55-1596-01A-01R-0946-07	3.383201951	3.603186022
TCGA-91-6836-01A-21R-1858-07	3.523812864	3.519381862
TCGA-49-4488-01A-01R-1755-07	3.491708701	3.579941098
TCGA-99-8032-01A-11R-2241-07	3.617048359	3.495515752
TCGA-49-6743-01A-11R-1858-07	3.570686571	3.45767039
TCGA-44-7662-01A-11R-2066-07	3.531099322	3.461020142
TCGA-44-2655-01A-01R-0946-07	3.422270361	3.573465471
TCGA-50-5946-02A-11R-2090-07	3.540534985	3.412371861
TCGA-86-8673-01A-11R-2403-07	3.5463303	3.53664878
TCGA-MP-A4TF-01A-11R-A262-07	3.529619342	3.464545754
TCGA-67-6215-01A-11R-1755-07	3.400686418	3.507402166
TCGA-55-7911-01A-11R-2170-07	3.315839155	3.483064641
TCGA-44-2666-01B-02R-A277-07	3.008414345	2.887963831
TCGA-97-7554-01A-11R-2039-07	3.521863803	3.408928435
TCGA-05-4426-01A-01R-1206-07	3.586973051	3.518305031
TCGA-97-8176-01A-11R-2403-07	3.485302696	3.548803675
TCGA-05-4382-01A-01R-1206-07	3.628320516	3.434154443
TCGA-J2-A4AE-01A-21R-A24H-07	3.452213246	3.466218205
TCGA-73-4670-01A-01R-1206-07	3.651919072	3.586762547
TCGA-44-2668-01A-01R-0946-07	3.630749685	3.432444907
TCGA-44-3398-01A-01R-1107-07	3.654479185	3.553107727
TCGA-50-6594-01A-11R-1755-07	3.44723549	3.574364623
TCGA-73-A9RS-01A-11R-A41B-07	3.390924553	3.584874313
TCGA-53-7626-01A-12R-2066-07	3.506575935	3.397357269
TCGA-55-8505-01A-11R-2403-07	3.682249142	3.541523488
TCGA-97-A4M5-01A-11R-A24X-07	3.483646438	3.478241211
TCGA-62-A46U-01A-11R-A24H-07	3.491402401	3.489480367
TCGA-44-2665-01B-06R-A277-07	3.136878607	2.78490583
TCGA-MP-A4TJ-01A-51R-A262-07	3.536254125	3.376873567
TCGA-62-A46V-01A-11R-A24H-07	3.388995648	3.558986069
TCGA-50-5055-01A-01R-1628-07	3.527703483	3.50493422
TCGA-69-8453-01A-12R-2326-07	3.574338863	3.459806107
TCGA-91-6829-01A-21R-1858-07	3.497961802	3.423754243

Table S4. Summary of 3682 genes in turquoise models

The genes in turquoise models				
RN7SL1	THAP11	ZMYM2	EXOC6	DHFR2
S100A6	IFRD2	CDS2	SNORA5C	ABI2
ACTB	VMA21	SIK2	YOD1	BAZ2B
ACTG1	DNPEP	TPP2	ERMARD	ZNF432
S100A11	ACOX1	NPRL2	MVK	ALKBH8
MT-RNR1	UBA3	OCLN	ZNF134	TTC21B
TAGLN2	AFF4	CHML	GNL3L	AC009237.8
IFITM3	SPRED1	KCTD9	AMMECR1	PDE5A
GSTP1	SRSF1	TTC13	FAM76A	MITF
CD63	CADPS2	YIPF4	STARD4	ZNF7
CALR	ZNF24	AC019117.1	CBL	GLIS3
LGALS3BP	APEX2	NUDT8	RRP15	CRYBG3
SLC25A6	ARHGAP12	CCDC50	ZFP90	RUFY2
PRDX5	IFI27L2	STK4	AP1AR	KBTBD7
HSPB1	RRM2B	TAOK1	ZNF888	HMBOX1
SNORA73B	FAM91A1	TOPORS	RAPGEF2	TNFRSF10A-AS1
PFN1	MGST2	ANKRA2	FBXL20	ZNF528
PPDPF	AP1G1	RAB13	GXYLT1	TDRD3
RPL36AL	ATAD1	ITSN2	SNORD100	CLCC1
PTMA	SLC30A9	ZCCHC7	SNX13	VGLL3
NDUFS5	NUBP2	MOB4	MLLT3	TBCK
ARF1	FBXW11	GATAD2B	CERT1	DCLRE1C
MALAT1	TP53BP2	SIN3A	UBE3A	ZNF823
GRN	FAM3A	PPM1A	ARID4A	GSTCD
P4HB	LZTS2	CRYBG1	RBM27	TCF4
SH3BGRL3	RNF25	CTTNBP2NL	APOOL	L3HYPDH
UBC	ECSIT	ELK4	PHACTR2	RNU7-49P
PKM	KLF9	ATG101	NUDT4	RNA5SP82
EDF1	EBP	GOSR1	SLC25A12	TRMT11
BSG	PTK2	MOB2	DSTYK	AL355001.2
EIF1	OSBPL9	KDM5A	RCBTB2	NEU3
SFN	EPS15	AEBP2	SNIP1	FBXO30
CHMP4B	WTAP	MED11	KLHL42	MFSD8
S100A16	ERBIN	RANBP6	ZNF397	POLK
GPX4	PCMTD2	BPTF	MOSPD2	AC139769.1
ATP6V0E1	YTHDC1	PCGF3	HPS4	RBM41
CAPNS1	TUG1	ZNF623	MIR3682	B3GALNT2
CFL1	PWWP2B	MPV17L2	AC099677.1	N6AMT1

KDELR1	SLC30A1	SLC49A4	FASTKD1	SPATA6
DDOST	FCHO2	BTAF1	AL513534.1	MTRF1
OAZ1	VEZT	TRIM33	PRKCH	NME7
XRCC6	ANKRD10-IT1	CCNYL1	CUTALP	PIGB
LMAN2	RARA	CDC73	PWWP2A	AC010542.5
ECHS1	ANO6	CUL5	DNAJC16	SNAP23
MCL1	TGFBR1	PLEKHA2	ARIH1	IPO5P1
CIB1	SPTLC2	GPANK1	FBXO42	AC015871.3
CSDE1	SPIDR	DIP2B	PDE12	CLEC2D
NDUFB11	RAB3GAP1	CILK1	ROBO1	ABHD18
BCAP31	TJP1	RBM18	CSNK2A1	RFXAP
SYNGR2	TNPO1	XPA	SESN1	FAM204A
GRINA	CPSF6	MAP2K4	CBLB	FAM160A1
SRP14	PRRC1	OSBPL3	GUCY1A1	KBTBD6
UBA52	CRAT	SMCHD1	MYCBP2	MBNL3
TUFM	AP3B1	EPC2	ITPR2	LONRF3
NUCB1	SPAG9	ABCA1	ATP11C	GANC
IFITM2	NBN	PBX3	GPATCH11	ZRANB1
PPIB	CXADR	TBC1D15	STAG1	ZNF558
FKBP8	EDEM3	MAGI3	MSH3	SLC10A7
TSPO	CHST14	DDX28	PCNX1	PIK3C3
ACTR2	FBXO28	TSPYL4	NIN	AC245884.3
POLR2L	TIAL1	RBBP6	TRIM13	SWT1
FLOT1	ENPP4	AKAP11	FAM126A	POLI
ERP29	ATMIN	ARL5A	CNOT4	TCP11L1
CD151	PSMD5	KDM1B	OMA1	OPHN1
PARK7	XIAP	ANTKMT	PHF21A	ZC3H14
SSR4	CLCN3	DHX36	SOCS7	RFX7
TMED9	PLEKHF2	TIMM22	IGIP	ZBTB26
NDUFS6	KLHDC10	HOOK3	ZZEF1	ZNF44
MYDGF	GMPPA	PLSCR4	RASSF8	KIAA1841
TOMM7	PCMTD1	FAM104A	STRN	ZNF615
NORAD	WDR13	UBE2I	MARK3	GTF2IP23
C6orf62	CTR9	KMT2D	PANK2	USP46
AUP1	R3HCC1	CAPN7	KIAA0753	ZNF101
SEC61B	FOXJ3	AVL9	PCNT	ZNF708
RTL8C	DNAJC4	VTI1B	MAPK8	CEP63
PLP2	SPSB2	ATE1	KIF21A	ATAD2B
SERINC1	ZNRD2	ZCCHC14	OTUD6B	DYRK3
SERINC2	EFCAB14	FERMT2	RC3H1	PTPN4
CD46	PPP4R2	RAB3GAP2	MINDY2	IFT88
NDUFB10	MGAT5	AHCTF1	DCUN1D3	SNORA3B
TRIR	PIP4K2A	SLC25A46	FAM13B	ZNF506

PPP1R14B	ACADS	RPL21P16	VPS8	NAF1
PIM3	CLPP	USP38	ZNF354A	ZNF197
MDH2	GALK1	NLK	NAA25	ANGPT2
LMNA	ATP13A3	ZDHHC21	PDE7A	MOSMO
APRT	CCDC115	WDR11	TMTC2	MPDZ
NUCKS1	SHISA4	EPB41L5	UTP25	GABPB2
MLF2	SLC35F5	KIF13A	DHRS4L2	NXPE3
CUTA	PCM1	ZNF106	ZNF398	OXNAD1
HSD17B10	AFDN	ZNF532	NCBP3	ESYT3
CNN2	GPD2	PGM3	ZNF254	ASTE1
TMEM109	AP3M1	HPS3	C18orf25	TMEM260
CDK2AP2	GHDC	ZFP62	ZNF267	ANKRD18EP
PCBD1	TIMM10B	HSD17B12	PPP1R12B	GPALPP1
BLVRB	BAG5	NSMAF	TULP4	THUMPD3-AS1
CAPZA1	DOLK	NAA15	INPP4A	BRWD3
SLC25A39	VPS41	PAWR	SPAST	ZNF586
SNORD17	TOLLIP	FANCL	WDFY3	NAP1L5
PMVK	SFT2D2	NVL	TBC1D4	GTPBP10
RHBDD2	CREBZF	CLIP1	S100PBP	LINC00205
PPP1CB	AGFG1	FBXL3	SLC9A7	TTLL5
ARHGDIA	NR3C1	PGP	IRGQ	GTF2IP4
TNFRSF12A	YIPF6	OSBPL11	MLLT10	PEAK1
DDX17	TMEM11	TRIM38	PPM1D	SLX4IP
HIGD2A	NCAPH2	PPP1R12A	TRAF5	PCNX4
PSMC3	PRKAR2A	HTT	IDE	NT5DC3
CHMP2A	PSEN1	DOK1	EEA1	NEK3
TMEM30A	C5orf24	NAV2	INPP5F	KIN
NEAT1	NFKBIZ	TXLNG	RBAK	LEF1
YIPF3	RBL2	NDEL1	WDR47	PLEKHA8
CYBA	SLAIN2	SENP5	FBXW8	ACVR2A
CLTC	PPP2R5C	UFSP2	ZC3HAV1L	WRN
MRPS34	AL390728.4	WDR33	EVI5	AP4E1
CNPY3	SEC24D	FAM120B	SLC12A6	HNF4G
CLTA	ANKIB1	GAMT	HERC1	ZEB2
CHPF	PPP3CB	SPG11	DNAJB14	LACC1
MRPL41	MTMR12	CCNT1	EPB41	PDP2
EFHD2	CUL4B	ZNRF2	RPS11P5	SATB1
TMEM219	FMNL2	TMCC1	CPEB2	CLMN
FLOT2	PHF10	DENND4C	DIP2C	USF3
ERGIC3	LANCL1	DCAF1	ZNF160	AC021078.1
UFC1	TICAM1	KATNB1	CDK14	THAP6
CLDN7	CAMK2D	CDK17	MRPL42	TTC33
VMP1	STX6	CACUL1	JMY	GALK2

PRKAR1A	RB1CC1	RAF1	CDC40	ZNF431
HIF1A	RUNX1	CCDC51	MON2	AC011477.2
PRDX2	GOLGA4	FEZ2	AC024060.2	TGFBR3
POLDIP2	SLTM	INTS8	BDP1	TET2
AP2S1	ISOC2	RNF115	FBXL4	FGF7
RRAS	LIMS1	ARID4B	DST	SGTB
UQCRC1	RAP2C	BCL7C	NR2C1	SLC38A9
TNFRSF1A	UFL1	SYNJ2BP	ZNF84	PLAGL1
AKR1A1	HIPK1	FAM160B1	DCP2	NKIRAS1
STT3B	RANBP2	ADNP2	RBM7	FAM133B
TRAPPC1	ORMDL1	EIF2AK3	NPAT	PYROXD1
RNF5	NTHL1	CBLL1	RAB33B	BBS9
MRPS18B	PSME4	RO60	TP53BP1	RBM48
VEGFB	ILVBL	LENG1	AC120053.1	DIXDC1
EIF6	SMURF2	MXD1	PRMT3	CENPJ
H1-10	ETV6	ADAT1	MECP2	AAK1
PPP1CA	ABCD3	TEX2	ITGB8	ZNF429
NOP53	SDE2	KMT2C	CEP104	RN7SL138P
NUDC	STUB1	RBM26	KIF2A	LRRC57
ELOVL1	JKAMP	ZNF266	CHD9	KCNA3
SHISA5	CISD3	PSMA3-AS1	DENND3	AL135844.1
SSNA1	AC083799.1	KLHL20	IRAK3	MTAP
PCNP	WWP1	CCNT2	NOM1	ZNF248
DPP7	CCDC117	PGM2L1	THUMPD2	RALGAPA1
MARCKS	ELOF1	GTF2H1	ARHGAP29	EMSY
BLVRA	TEX264	GAS2L1	RNF103	AKT3
SLC25A1	ATF2	NSD3	TEX10	ZNF420
DRAP1	PYCARD	PRPF39	ZNF28	SMG6
SCAMP3	CCNL1	DICER1	ZNF544	MIR5581
ARRDC3	SCYL2	PFKFB2	ZSCAN29	AC025165.5
RTL8A	PRPF4B	PIGBOS1	ZCCHC8	SVIL-AS1
CAPN1	NCOA3	ZYG11B	MYNN	LCOR
CYB5R3	ZNF207	PTPRM	LZTFL1	ALMS1
AAMP	SEPTIN7	RPS6KB1	ZNF761	XAF1
TXN2	PTPRK	ACAP2	DPY19L3	AL139396.1
SFPQ	U2SURP	BBX	FAM114A2	TEFM
ATP6AP1	SLC11A2	GLCCI1	KIAA2026	PAGR1
TMEM9	ANKRD40	INO80	THADA	TTC28
PHPT1	TAF9B	IREB2	AGGF1	ZNF510
RNF187	CCDC22	THUMPD3	PDPR	TMEM161B
RAB1B	NOTCH2	HEATR1	UPRT	SLC16A7
AIP	SUCO	BCL2L11	MAK16	ZNF548
FBXW5	CSNK1A1	SPATA2L	KLHL8	PMM2

SNU13	RESF1	GABPA	CCNJ	NUBPL
KIF5B	ZNF524	MIR186	PALB2	L3MBTL3
KRTCAP3	SH3RF1	FBXO33	CHM	AC011468.1
FAM50A	NAT14	NCK1	TNKS	BCLAF3
QARS1	ANKRD13A	YLPM1	ZNF680	COL21A1
UBALD2	LONP2	UEVLD	RSPH3	SLC2A13
AHNAK	DOCK1	ZNF609	ENTPD7	CCDC134
UBXN1	MIA3	VPS13C	ZSCAN32	TOGARAM1
ATP6V1E1	MPZL3	ZBTB44	MBTPS2	MARCHF1
AP1M2	CSGALNACT2	DCP1A	TET3	PCBD2
LMAN1	DMAC2	EXOC5	TRIM23	AL031282.1
UBXN4	DPCD	ZNF260	AC007485.2	IKZF2
YIF1A	AL049840.5	LNPK	NGLY1	AL512791.1
CENPB	PDLIM5	SDCCAG8	RIC1	PLEKHA3
RBM42	NFKBIL1	KDM5B	CCNH	PRKAG2
SDF4	PHETA2	NHLRC3	PELI2	ZNF43
FAM32A	NCKAP1	CCDC186	AL592295.6	SEC61A2
IQGAP1	MPST	ZBTB41	RNASEL	NSUN3
ECH1	RNF146	TM2D1	ZNF518A	ZNF124
UBE2M	PPIL4	TMA16	RCHY1	SMYD4
PPP1R14BP3	RITA1	TCF7L2	TRPM7	PUS7L
DDRGK1	DYNC1H1	MED28	NEK4	ZNF550
PNRC1	THUMPD1	KLF11	ATXN2	TRPS1
ATP5IF1	ERAP1	MRPL19	HMG20A	TANGO6
VWA1	FAM241B	ERN1	THAP3	AC073857.1
PICALM	PDP1	COG3	GXYLT2	CD302
RNF167	BMI1	MCM3AP	DYM	USP8
STARD10	SPPL2A	IFI44L	ESS2	ZNF671
PPP1R11	SNAPC2	IL17RC	IQGAP2	SLF1
MFSD10	GMFB	VPS26C	METTL14	TMEM102
GFUS	SH3D19	ELMOD2	NEMF	ZNF354B
CREB3	CDC27	SNX19	ASB7	AC004839.1
SLC38A2	PKN2	MAP3K1	MTF1	AC092070.2
DYNC2I2	CH17-340M24.3	EIF2AK4	ZC2HC1A	REPS2
SDF2L1	CCNC	SCRN3	BORCS6	NEDD4
IRF2BP2	ANKRD17	NSD1	GULP1	INO80D
PHC2	MPRIP	BTBD3	ZNF511	HSPBAP1
R3HDM4	AC005332.3	FBXO38	AC092747.4	LINC01004
FAM120A	MAVS	DMTF1	PLN	AL158166.1
TMEM54	C5orf51	RUFY3	ZNF841	SEMA5A

RAB34	ARFGEF1	ASCC3	AC093297.2	ZNF720
CUEDC2	EP300	CNOT6L	KCNQ3	ZNF600
EDEM2	TRA2B	TBC1D5	FBXO3	ZNF462
PPP2R1A	SLC30A5	ATP8A1	EXPH5	KLHL15
CD47	MTREX	SAMD9	ZNF641	AL035413.1
CTDSP1	PPTC7	MORC3	SASH1	MFSD14C
MAT2A	SETD5	CNOT2	ZNF252P	ARID1B
TLE5	TMEM222	ST8SIA4	EPG5	PTPDC1
RNF11	NUMB	ZBED4	MIR4263	AC011477.1
RAB14	SRSF10	RAPGEF5	SNRPGP15	MAP9
SF3B1	RPUSD1	ACD	POC5	RPL32P3
SPR	MPV17	KDM3A	ARHGAP42	ZNF595
EMD	USP33	RELL1	IPMK	EDRF1
PEF1	RBM25	ZNF121	ZNF184	ELP1
ARL8A	WDFY1	PRKAB2	CDK6	ZNF256
CAPRIN1	SLC66A2	SPOPL	WWC2	BORCS5
RAD23A	TMEM106B	ZNF148	YPEL2	SCML1
MSRB1	TLR2	PTPN2	SENP7	BRCA1P1
ARL8B	PURB	CCND2	USP3	ZNF180
CCDC124	CHSY1	STEAP2	MTMR2	PRKCE
KLF6	LARP4B	DENND6A	TTC14	APTX
DCXR	MVB12A	RNF170	BRWD1	TTC5
RHOC	BMPR2	TRRAP	HEATR5A	BX537318.1
VPS28	SEC24B	RPS6KC1	ELOVL7	ZNF136
PGAP6	SEC23A	CEP350	UHRF1BP1L	PLCL2
HCFC1R1	CMTM4	FLJ20021	RNU6-418P	CEP120
TMEM214	RGL1	SECISBP2	PXK	HCG18
GIPC1	DDA1	ANKRD13C	PIAS1	ZFP69
METTL26	RFC1	KIAA1143	SSH2	ADGRL2
IPO7	GRHPR	SOCS6	C21orf91	SNORD19
WBP2	WAPL	ACTR8	CEP192	SNORD69
DNASE2	ENTPD4	UBA5	ZNF608	FAM185A
ZFAND5	AMD1	AC069282.1	LCLAT1	AC060766.1
CDIPT	SGMS2	PDZD8	RAB11FIP2	CCDC107
TST	KMT2E	NUDT22	ZBTB43	RNU4-47P
LMF2	ZMYM4	TRIM5	NUP42	INVS
ARPC4	MED13	PIGA	NAGPA	ZNF346
CLPTM1	RABGGTA	GTF3C4	MEF2C	AC079140.2
DGCR6L	OSGIN2	TCEA2	RELCH	PRDM10
SIGMAR1	OCEL1	MAP1B	UGGT2	MYO9A
MOB1A	UNC45A	TKFC	MED13L	AC136475.10
RALY	FEM1B	COG5	PDPK1	ZDHHC23
CNPPD1	ARHGEF12	WDR48	MAP3K4	ZNF484

PNKD	TANK	JRKL	DIP2A	GABPB1-IT1
EIF3G	PEX13	SS18L1	AC107983.1	PIK3CA
TMED7	ARFGEF2	TMEM47	ZMAT3	ERI2
ITGAV	IL33	TRAPPC8	SHQ1	MCPH1
RGS1	DHX29	REST	RBM43	ZNF737
SEC31A	N4BP1	FOXN2	ZNF621	DISP1
BTBD6	SCAMP1	UBR3	POLH	AC015813.6
ACTR1B	RAP2A	VGLL4	ARL13B	BCAS3
GNG12	EIF2AK2	APPBP2	ARHGAP26	TBC1D12
DNAJC3	SLC35C2	THAP5	FRK	TBC1D8B
ZNF664	CYB561D2	UBA6	CYP2R1	ZFP3
NME3	G3BP1	PRDM2	FRYL	TSPAN2
ADPRS	NT5C	CHD6	HERC2	TNRC6C
TMEM167A	VPS54	CPEB4	ATXN1	ZNF100
CHCHD10	OXSR1	AGO4	RBM15	AL355488.1
COPS7A	BCAR1	RIPK1	MCTP2	CDYL2
TMEM208	MRPL23	SGF29	TUBD1	LRRC8C
ACBD3	SLC30A6	STIM2	MYO5A	TAF4B
NME4	DPYD	SESTD1	KAT6A	PLEKHM3
COPE	KLHL24	WEE1	ALG6	EID3
NINJ1	HPS6	MPND	KANSL1	FRY
CORO1B	SNORD94	TRIP11	WDR20	ZNF845
GTF2F1	AFF1	KLHL23	ZNF292	RPS12P3
DPM2	TACC1	MINDY3	TMEM106A	LMLN
IGSF8	TMEM161A	EXOC8	TAB3	LRIG2
UBE2D3	LHFPL2	LATS2	SUGT1	TSTD2
AGTRAP	LARP4	TUT7	PDLIM3	HELLS
USO1	NR1D2	ARFGEF3	PLEKHG1	BRAF
ERG28	RIN2	RABAC1	PURA	ZNF718
ITPA	TENT2	AGL	SLC4A7	NMT2
TIMM13	ZNF638	CDC37L1	ABL2	AC027644.1
SLC2A4RG	OSBPL8	ABHD17B	SNX29	SFMBT2
ACTR1A	RHOQ	HPS5	FBXL17	AP006621.2
HM13	LIN7C	ZNF117	APOLD1	DTWD2
STX10	SCAF11	PPFIA1	PTPRG	HDGFL3
LTBR	FGL2	PAG1	LONRF1	AL121839.2
LEPROT	CCNG2	EOGT	HIVEP1	CHIC1
TUBA4A	SERTAD2	CUL3	HECTD4	DOP1A
TSR3	PEX16	ZBTB10	NAPEPLD	ZNF607
DNPH1	IBTK	MTF2	GAREM1	JRK
SLC35A4	RCOR1	TAPT1	CEP68	FAM111A-DT
HNRNPU	UTRN	CLIP4	ZUP1	ZNF678
POLR2E	EIF4G3	ORC2	MTX3	PARP15

ARPP19	MLST8	PIK3R1	CYP20A1	ANK3
PJA2	ADCK2	ZNF302	FAM98C	MPHOSPH9
FGD5-AS1	GATAD1	MERTK	HERC4	AC137932.1
PSMC5	TRIO	FAM193A	MIR4635	DSE
MAPK1	DCAF5	CARMIL1	DDX60L	FAM200B
AKR7A2	TFPT	RNASEH1	CRBN	GPATCH2L
ARAF	SEC23IP	ESCO1	BMPR1A	ZNF551
PNRC2	ARHGAP35	MDM4	PSD3	ZNF417
CAB39	CBX5	PIBF1	KANK1	SMAD9
TRAPPC6A	STRN3	GON4L	ASB3	CPLANE1
IL6ST	MVD	WDR36	TRAPPC13	ARL6
RNH1	EMC2	MEX3C	THOC1	ZNF37A
PTPA	AKAP13	TRIM32	AP5M1	BTD
SERF2	TEAD1	SBNO1	APH1B	AC024075.1
ADRM1	CNOT6	SNAPC3	ZNF75D	STARD13
GFPT1	TBC1D9	USP54	SIX4	IFT74
PKP3	RNF141	NUDT18	SNORD46	RN7SL834P
EIF5	GNPTAB	RBSN	EVI2A	RUSC1-AS1
RETREG2	TMOD3	CDKAL1	SEC22A	ZC3H12C
SPCS3	FAR1	C2orf49	FRMD4B	SNHG4
C9orf16	COPS2	FOXN3	ZBTB21	SIAH1
ZNF622	DCAF16	DCAF10	USP42	ZNF69
COASY	RBFOX2	GCDH	TMEM135	MPP6
PTPN12	RABGGTB	TAOK3	PMS1	MIR5587
DTX3L	UBR5	CHUK	CEP170	RGS17
ACSL1	KLHL5	SMCR8	EXOC6B	ATP9B
NDUFAF3	NPEPPS	ANKRD54	VPS13B	ZNF585B
GSPT1	TBC1D23	DPY19L4	ME2	ATF7IP2
CTSA	RMND5A	ANKRD28	ALS2	TERF1
PUM2	MBOAT2	ALDH16A1	YTHDC2	ERICH1
SEC13	PAFAH1B1	MIOS	HLA-DQB1-AS1	METTL15
AP1B1	GMCL1	RLF	PER3	NEK1
KIAA2013	YJU2	VAMP4	GCFC2	USP46-DT
PFDN1	MACROD1	MOB1B	JAK2	CDADC1
TOP2B	DDX58	SYNRG	ZBTB14	LINC02035
RUVBL2	DENND5A	MIS18BP1	STK3	KATNAL1
BAX	ZKSCAN1	RCOR3	CIAO3	ZNF280C
SLC25A11	BLZF1	PDIK1L	HLCS	ZNF234
EIF4EBP2	EXOC4	AKAP9	ZEB1	ZNF250
SHKBP1	TRIM44	NADK2	POC1B	NSUN7
SPTBN1	RBBP5	ZNF408	APAF1	PIGF
GLS	VHL	PLA2G12A	C2CD3	AL353743.1

OGT	DENND11	AC018645.3	CCP110	PLD1
FBXL5	TRAK2	TMTC3	CHORDC1	LCA5
ABHD11	ITFG1	SYNE2	MIGA1	ZNF430
SCAND1	PRPF38B	GOLGA1	SLC35B4	TIAM1
ERAL1	MAP3K5	RASA2	CASK	BCL2
SZRD1	ZC3HAV1	AP003392.2	IPO11	SSBP2
APEH	SLC12A2	SNX24	FAM76B	PDZD2
PTBP3	PTEN	TRIM25	NFAT5	FCGR2C
CD2AP	CAMSAP2	R3HCC1L	ZNF14	RNU7-40P
TMEM165	LRRK2	ARL15	CNTRL	GDF11
WDR82	MACC1	LARS1	ZNF140	DIMT1
GALE	PHACTR4	RCAN3	ZNF766	ZNF606
CLINT1	PLEKHJ1	FLT1	XPO4	GPM6B
UHMK1	RHBDD3	NOC3L	ZNF776	LINS1
WASL	USP16	SEC22C	GLRX2	PIGCP1
IDH3G	RNF6	RC3H2	TBCEL	AC131009.3
MAP7D1	PHKB	NKTR	CCDC125	ZNF567
KCTD12	RAP2B	KDM7A	AC126474.2	ARSK
GPR108	RSPRY1	ARHGEF7	COG2	SWSAP1
RHOD	EXOC2	OGFRL1	SLC39A3	ALDH1L2
TMEM203	MARF1	FDXR	ZNF516	AL135905.1
EMB	FAM168A	UMAD1	AC027117.1	FAR2
MRPL20	FCHSD2	DHRS7B	RAVER2	ARHGAP10
CPNE3	RALGAPA2	DALRD3	CYP4V2	GLUD1P3
PBDC1	YIF1B	FAM217B	Z99129.4	PACRGL
PFKL	WASHC4	ATG12	PHTF1	FOXP1
PHLDA3	SDSL	DYRK2	HELQ	RALGPS1
REEP3	FAM207A	ANKRD49	TUBGCP5	PAXIP1
AHR	EEFSEC	PRKD3	MAP2K5	STOML1
XPR1	KLHL2	GARRE1	VWA8	SGK3
YAP1	EMC4	TEP1	ZNF669	AC102945.2
PPP1R15B	TTC19	BLOC1S5	DZIP3	HGF
RBCK1	TMEM184C	TANC2	AASDH	INTS6L
NR2F6	ADAM10	H2AC20	SNRNP48	PMS2CL
IVNS1ABP	NUS1	CRLF3	AC144530.1	ZNF736
UGCG	SEC24A	NDUFA5	LINC00667	TBC1D19
USF2	NIPBL	KPNA1	MBP	KDM6A
MAPK3	SGMS1	SMARCA2	ZNF700	LINC00641
BRMS1	CDK5	CCDC93	IL1RAP	PARP11
OGA	RYK	LMBR1	CEPT1	FUT4
UBE2K	CASD1	SMAD4	MIR559	PAQR3
ANKRD10	ZNF655	GIGYF2	MDN1	CEP97
UBL7	GOPC	RREB1	MGA	ZNF649

C11orf68	BCL2L12	ZNF875	FGD4	CCDC82
SAT2	ZC3H7A	KAT7	WDR5B	SLC22A15
KEAP1	SRPK2	BICRAL	ARID2	PRKAA2
ABHD12	OIP5-AS1	MANBA	AGK	ASH1L-AS1
CREB3L2	C1GALT1	RBM33	MYSM1	AL449212.1
RELA	RABGAP1	KLF7	ZNF880	ZC3H8
ATP5F1D	NUP43	PHIP	MKRN2	RIC8B
NEK6	RSRC2	SYTL2	KRR1	CEP162
API5	TMEM187	SNHG16	CRYZL1	CEP83
YIPF2	INSR	TMEM168	ZMAT5	DSTNP1
TMEM120A	MACF1	CDPF1	APBB2	ITSN1
EDEM1	NFX1	GPR34	CDK13	ST3GAL6
DDX3X	RAB27B	NT5C2	PHLDB1	CEP112
SLC10A3	ITPRIPL2	STRBP	COBLL1	SUMO1P3
SSR1	SLC19A2	LPAR6	CCPG1	EXOSC3
PAFAH1B3	ABRAXAS2	ZBED5	SLC33A1	AL021707.8
MPDU1	SETD7	FOCAD	GLMN	ANAPC1
MBNL1	RBM14	CHD8	C20orf194	ZNF697
ATP6V1A	LNX2	LRP6	SNORA71B	ZNF780A
E2F4	MMP24OS	MED23	ATXN7	KCNJ2
TADA3	NCBP1	ARHGAP5	DYRK1A	RASGRP3
TC2N	RBMS2	CHD1	ZNF182	ZNF675
RPS28P4	MAP4K3	HEATR5B	HCG11	PARD3B
ADSS2	CREBBP	SAP30L	ZNF721	ZNF792
GPBP1L1	SLC41A2	CARNMT1	PDK3	ZNF626
EFR3A	AMMECR1L	CERS6	VPS50	AL137003.1
PARP14	TIMM29	CCDC106	CRYZL2P	ADAMTS9
MRPL55	KHNYN	ZZZ3	TLR1	INE1
UFM1	GGNBP2	PRAF2	LRCH3	MIR320E
SON	LINC00674	EYA3	ZNF45	MON1A
UBQLN1	TASOR	FUT11	ZNF25	DTD2
PRRC2C	LPP	CEP57	USP15	PUS10
MARCHF6	RAB22A	C2CD2	KIF3A	ZNF529
GPS1	AKIP1	TMED1	PRPF18	AC025171.1
CD276	TMEM39A	SOCS4	INTS12	ZNF846
PARP4	FAM199X	DDI2	ZNF264	AC127024.4
RNF19A	RASEF	DCUN1D4	MPP5	TEC
MFSD14B	MIR570	EPM2AIP1	TRMT13	ZNF268
RNF13	CHMP6	ZNF561	FAM89B	C8orf44
RAB5C	PDCL	C2orf69	ZXDB	ZKSCAN3
HP1BP3	PCGF5	FAM98B	DBT	C16orf87
HUWE1	SMAD5	RABEP1	NAPB	MTMR9
MCRIP1	TTC17	ZKSCAN8	ZFYVE26	ZNF445

SH3GLB1	ARID5B	MTR	PPP1R9A	PIGN
ELMO3	USP12	MRM3	TARS3	RNF217
SAR1A	C6orf120	SAMD8	ZNF507	NIPBL-DT
ABI1	SETX	UBXN7	ZMYM1	AC008115.3
GSDMD	SERINC5	DNAJC7	MAPKAPK5	AC005104.1
PQBP1	ACACA	RNF111	ZNF41	METTL8
RBM47	RBMS1	DECR2	SFMBT1	SUPT3H
UBXN6	UPF2	ZSWIM6	NFXL1	SCAPER
TMEM179B	GTF2A1	BBS10	INPP5B	TMEM154
BAZ1B	BLOC1S6	IRAK4	ATG2B	AC010834.3
MRPL10	NAPG	AGO2	NR3C2	FAM241A
STX5	ATF7IP	MECR	FARP2	AC026436.1
PUM1	RASA1	PTDSS2	ENTPD5	ZFPM2-AS1
SPRYD3	RAB2B	LRRCC1	PGGT1B	TCP11L2
NDUFS8	C6orf226	RNASEH2C	PAPOLG	ZNF780B
ELF1	HBP1	FASTKD2	ZNF704	TUBE1
BCL7B	PLXDC2	ITGA1	DIAPH2	SHLD3
THAP4	PSIP1	LNPEP	PBX1	ZNF605
MCRS1	SERPINB9	TMEM170A	GPR180	AL353807.4
SPIN1	LYSMD3	CWF19L2	SMAD2	ZNF224
FPGS	APPL2	RNF144A	TRNT1	FAM184A
MRPL28	TRIQK	RHBDD1	PTPN22	RNF125
STX12	BACH1	FAM214A	MALT1	C8orf37
UBL3	OXR1	TNRC6A	PKNOX1	ZBTB25
DHX15	RB1	FAM13A	ATP10D	ZNF10
SEL1L	ITCH	KAT2B	ZFP1	USP31
BPNT2	SNX14	WNT5A	LINC02257	ARHGEF9
DNAJB2	ERV3-1	PTPN3	TTC39B	TMEM67
DDX41	COMMD4	UTP23	MLH3	GPR155
CDC42SE2	TMED3	LTN1	ARSB	REL
TMEM263	CHURC1	ANGEL2	LIN54	PRR34-AS1
ZDHHC20	HSD3B7	REV1	CSNK2A2	IFRD1
BCKDK	UBP1	CIPC	DENND1B	ZNF611
MYO1B	ASH1L	FBXL15	PDE4B	PDE3B
PELI1	MTMR4	C19orf25	DENND4A	ZSCAN12
SSH3	C4orf48	PHTF2	PEX26	ZC3H6
TIMM17B	TLK1	GCC2	KLHL18	ATP6V0A2
PES1	ZFAND2B	LUC7L2	SLC36A1	TSEN2
EML4	THOC2	UIMC1	LRCH1	RNF216P1
RBM39	ZBTB33	TMEM65	DOCK10	DENND5B
WNK1	PPP2R5E	GAPVD1	SNORD101	RNU6-415P
BROX	SNX18	HUS1	ZNF253	ZNF557
DESI2	SMIM22	UBR1	FAM102B	NEMP2

ALG3	MAN1A2	CREB1	TRAPPC2	CPED1
CHID1	BIN1	HELZ	LINC01023	ZNF227
IMP4	BNIP2	TRAF3	ZNF587	YAF2
SCARB2	BCL10	ZFC3H1	ZBTB24	NAALADL2
ELK3	DDX60	FAS	SCO1	CLCN5
OTUB1	PPP1R21	ABHD13	JAZF1	PHOSPHO2
PAFAH1B2	MED14	SLC25A16	POGLUT1	TFEC
KITLG	TCF20	SNORD6	PEX1	AC010761.1
TMEM115	CFLAR	ZBTB5	Z83843.1	ZNF597
CCDC6	ATP11B	ATG4C	SYNJ2	ZNF391
PLOD3	PNISR	IKZF5	RPL23AP64	AC036214.2
TOR1AIP1	RAB8B	XRN1	NSUN6	GRAMD1C
ARPC5L	CCDC12	KRT7-AS	PDS5B	ZNF141
CAST	SLC25A24	MTM1	RUNX2	ZNF300
TP53INP1	FNIP2	RPL13AP20	LRRC37A16P	SGCE
PGLS	SLC35B3	ZBTB11	SP4	ROR1
HMOX2	XPO1	TMEM53	CSNK1G1	FER
PPP1R7	NCOA2	CCDC14	NAV1	AC000123.1
FNBP1L	NUP50	TAF1	USP6NL	ZNF714
FLYWCH2	CDC42BPA	RAB21	KLHL28	AC084824.1
NR1H2	ROCK2	CPNE8	SAMD4A	STON2
CD320	FOXO3	SPIRE1	AL662795.2	AC007996.1
FIBP	UBXN2B	DAPP1	AP001052.1	FMN1
RIC8A	NCOR1	TMED8	FAM149B1	ZNF559
ZMYND11	GPR137	RNU7-45P	C2orf42	ZNF485
OSBP	VPS4A	UTP20	ARHGEF38	AL928654.4
PDCD6IP	THAP9-AS1	KIAA1671	TNIK	DOCK7
C20orf27	DDX46	STX2	LINC01137	CYCSP34
NUDT21	SNX25	SSH1	G2E3	FAM120C
TSEN34	CEP70	AASS	ZFYVE9	AL031985.3
COMT	KANSL3	PCF11	LARP1B	AL353763.2
GTPBP6	AGPS	PHF6	RASAL2	CAMKMT
ZDHHC12	RAB28	PPP3CC	DCAF17	ZNF449
TMEM129	POLR1F	ARHGAP32	AC048341.2	METTL25
SORT1	ARMH3	SNX27	CCDC18-AS1	TMEM170B
GOLM2	CNOT7	PARD6B	PRMT9	Z98884.2
ITGA2	CHMP2B	RPUSD3	C3orf52	ZNF543
PAPOLA	PPIG	NTMT1	ZSCAN25	PSMG4
TNIP2	PIK3C2A	DIS3	SUZ12P1	STAT4
C11orf24	SRPK1	CYLD	NBPF15	AC084036.1
GNPTG	EBLN3P	RGP1	NSL1	ITPR1
NDUFA3	TCF12	FAM172A	KCTD7	MCTP1
PCIF1	CCSER2	PRR14L	CLASP2	ZMYM6

MYO6	CHASERR	OTULIN	ZNF320	SNORD83A
TRIP12	BABAM1	ZADH2	ZNF654	PIAS2
TMEM167B	RGS14	PLCH1	ENTPD1	CA5BP1
PRKAA1	TSPYL1	KIF16B	RAD54L2	ZNF808
SLC20A1	INIP	UBAP2	MTMR10	RAB30
ZNF217	ZNHIT2	ZNF800	ARAP2	ZNF181
PIH1D1	NFIB	GORAB	ATM	FUT10
MAPK1IP1L	TAF1D	SMG1	SEPSECS	DPY19L1P1
CRB3	UCHL5	KIF1B	GAB1	AC006504.7
RIT1	KMT5B	SLC4A4	CYB5R4	AC020916.1
AC034236.1	SMC5	TOR2A	ZCCHC4	AL031775.2
NRP1	CELF2	VRK2	CCDC68	ZNF37BP
FUBP1	ICE1	MSANTD4	DOCK4	ABCA5
MARCHF2	SETD2	AP1S2	PLEKHA1	HMGN1P36
TMEM41B	FAM111A	ZNF83	SNORA11F	ZFP30
ATL3	HIPK2	SPAG1	KMT2A	MTO1
TAB2	EDNRA	MANEA	UTP15	SLC24A1
SOAT1	COMTD1	ZNF552	PCDH17	WDR92
KCTD20	HAGH	PHF3	KIAA1586	WDR31
CHMP1A	RNF138	MIER3	GCNT1	ATR
FAM174C	UBR2	LMBRD2	MTERF4	ZNF200
CCDC85B	RNF38	ARL14EP	SHROOM4	AC135050.5
MBNL2	TTC37	SLC25A40	RASGRP1	ANKRD6
NSDHL	HNRNPLL	RNF168	ZNF35	ZNF461
C1orf122	MARVELD2	ATF7	ATP7A	SBF2
RAB5A	AIDA	MLH1	CREBRF	NUDT13
RAB18	SWI5	FOXO1	ZNF816	ZNF236
HDHD3	MTMR6	CRIPT	EHMT1	INTS6
NEK7	MIF4GD	ANKRD12	SNORD62B	CCDC102B
ATP6V0D1	PIN1	PARP8	EPB41L3	CC2D2A
STK17B	STAM	LATS1	ZNF587B	RPL22P24
BET1L	SLC25A36	PHC3	FBXO25	ARHGAP24
TOM1	STXBP3	ASXL2	CHD7	AC024580.1
YTHDF3	TWF2	PPFIBP1	DMXL1	PNRC2P1
KIAA0100	TARBP1	PARG	ZNF326	AGO3
PPP3CA	TRAPPC11	VCPIP1	PEX11G	BBIP1
GNA13	USE1	ANKFY1	NABP1	AL078581.1
GOLIM4	USP25	ZNF770	ZHX3	ZNF789
IFNAR1	TPRA1	UHRF1BP1	SLC36A4	RAD51D
SP1	GRWD1	LRRC8B	MAGI1	P2RY14
FBXO6	UBE4B	PTAR1	AC004918.3	ZNF790
ZRANB2	RNF144B	QSER1	SNORA66	AL606834.1
RNF213	MTMR1	CLOCK	LAMA2	AC084824.5

PPP4R3A	ZBTB1	USP34	ZNF75A	GEN1
RPS19BP1	RHOT1	DIPK2A	KDM4C	AC012467.2
ADNP	SOS2	GMPPB	OBI1	ZNF772
SIPA1	LGALS8	RABL3	MRRF	AL121772.3
RNPEPL1	ZNF12	MAP3K20	SCYL3	PLCE1
ECPAS	SLC39A10	USP13	AL132989.1	MEIS1
TBL1XR1	RAD17	MRTFB	SPRTN	PCDHB14
SMARCA5	KLHL9	GSAP	NFATC3	CCT6P3
JAK1	ETNK1	NRF1	OTUD6B-AS1	CCDC66
MFSD5	TMEM131	AOPEP	DMXL2	ZNF566
MPG	OGFOD3	HIF1AN	OTUD3	ZNF443
RNF149	TMX3	BCKDHB	ZNF304	LSAMP
NAGLU	UBR4	SNORD89	LSM8	ATAD5
RAP1A	CHD2	STX17	INPP4B	USP45
ARMCX3	ZBTB38	RALGPS2	TRMT10B	CEP135
PI4K2B	ARMC6	FKBP14	ZNF614	STAMBPL1
SPAG7	RNMT	PDE8A	AP003392.1	PRRG1
LIMCH1	PSPC1	GNB4	AC015849.3	AP4S1
MAN1A1	KIAA0232	POMK	ABHD14A	ERCC4
DDX6	HOOK1	MAML2	MAP3K9	DDHD1
ARSA	SVIL	COA1	ZNF107	PCDH18
GPBP1	SNORA33	B3GNT5	ITGA4	NBPF12
VEZF1	KXD1	SLF2	TENT4B	EP300-AS1
WDR26	SPART	ZNF436	XPNPEP3	AL031673.1
BTBD1	NSRP1	CRTC3	FAM135A	MCC
OXLD1	APPL1	DYNC2I1	BNIP3P11	AC016394.2
PYM1	FMR1	ABHD5	WDR19	AC132942.1
RCC1L	SAR1B	TBC1D14	SLC38A6	ZNF280D
YES1	SESN3	TLK2	PPM1K	MIR378J
THOC6	MED1	DPP8	POLR3B	AC090198.1
RANBP9	SACM1L	RABGAP1L	ZBTB34	HAUS3
SS18	SLC35A5	LRRFIP2	PHF20L1	TLR6
STAG2	FGFR1OP2	ELF2	SLC25A51	EXOG
RRP9	STAM2	PLPP6	FRA10AC1	PLCB1
TMEM101	SEC22B	NR2C2	FRG1HP	ZNF827
MTUS1	MAP3K2	ANAPC4	GFER	AL133371.2
TECR	RYBP	GRPEL2	IFT80	DNAJC18
GPCPD1	KDSR	SLC35D1	WDR35	LRRC37B
CTDNEP1	LRBA	SNRK	RBM12B	AL355574.1
PHF23	ASAP1	DOCK11	CAPRIN2	AC093752.1
RAB12	MEF2A	CMTR2	EIF4E	POLA1
SLC39A9	ETV3	RBMXL1	PLEKHH2	ZNF347
PPP4R3B	B4GALT7	FLVCR1	ELAPOR2	RORA

HIPK3	SLC30A7	ZNF143	SLC35A3	RGMB
NMRAL1	NAB1	SIPA1L1	APC	ZNF525
MFSD6	RMDN1	ZNF131	RNU6-850P	RNU6-762P
ATP6V1C1	ZNF644	VCPKMT	ZNF195	ALG10B
CBFB	MRPS30	AGO1	ITGB3BP	DGCR11
VPS4B	LRRC40	BMP5	ZNF451	AC145207.9
FBXO34	RCBTB1	NOL9	PHLDB2	ERCC6
HYAL2	TMEM160	ZNF791	PRIMPOL	PCNX2
ECI1	ZNF189	USPL1	EIF4E3	SLC7A6
MARCHF7	FEM1C	HIVEP2	CRNDE	GAS5-AS1
TRIP10	SP100	RSF1	CTNND1	ENDOG
RPS6KA3	PTER	MMAB	GK5	ZFP14
USP7	STYX	SREK1IP1	RBM4	RPL39P36
SYNCRIP	TMEM238	STX7	ZNF350	ZNF132
CNOT1	GPATCH8	PPHLN1	PDK1	AL162274.2
HRAS	NRIP1	PDE4D	BBS7	ZNF441
ELL2	ZDHHC2	ZDHHC24	RNF169	PTBP2
SIL1	NCOA1	MED6	ING3	SPATA7
RPS6KA4	MSL2	RRAGB	GPATCH2	RUBCN
MDFIC	SIKE1	RICTOR	ZCCHC2	AL035587.1
BAK1	USP48	NAA35	ALPK1	GIT2
CACFD1	SIRT1	GZF1	PTPRB	FRMD5
PRKCI	SP3	N4BP2L2	FBXW7	IL18R1
BIRC2	AC005261.1	ZNF652	CENPC	RPAP2
ORAI1	EEF2K	ERC1	GDAP2	DNAJC24
SLK	ARL5B	PAXBP1	C9orf72	GIN1
GNAQ	MIB1	CTDSPL2	PCCA	GTDC1
LPGAT1	SPTY2D1	TANC1	ZNF91	SPATA5
YDJC	CFAP97	WDR41	EPN2	FBXL2
RBPJ	PIK3R4	GRK3	PCDH7	CBWD2
CGGBP1	USP24	MSI2	HOMER1	LRRC37BP1
KDM3B	RPS6KB2	USP53	OSBPL10	ZFAND4
TMEM87B	DR1	NAA30	TLCD4	B3GNT10
GOLGB1	MIER1	ACER3	ZNF518B	ZNNT1
REEP4	MZT2A	AMOTL1	ETFB	TRAPPC6B
LRPAP1	KIDINS220	LPIN1	TMEM144	BIVM
G6PC3	MYO5C	UACA	CALM2P2	CCDC88A
PDS5A	MDM2	BTRC	ULK2	AC138207.4
COP1	FZD5	DAAM1	INTS2	RPGR
TRABD	PATJ	TSC22D2	AL049840.2	LINC01184
NUFIP2	CAAP1	FNIP1	ETAA1	SLC22A5
TNKS2	GUF1	ZFX	MIR590	XRRA1
CEMIP2	LRRC58	TTPAL	SETDB2	AC024075.3

HPCAL1	HS2ST1	USP47	PPIP5K2	HACE1
MOSPD3	RALGAPB	TMX2P1	SNX16	CDC14A
TWSG1	SNX30	NHLRC2	EPC1	MYEF2
PPP6R3	COX15	SIK3	FBXO22	TRPC1
SKIL	PTPRJ	ZNF92	CASP8AP2	ZNF407
ZFP91	LHPP	PRKCA	TNRC6B	ST7L
ESRRA	VPS36	ATRX	N4BP2	AC073046.1
JOSD2	DNAJC13	ZNF33B	AC010186.3	ZNF85
TIA1	MAN2A1	PPP2R3C	MAP3K21	KLF12
FAM8A1	SCAF8	RMND1	SYNE1	AL391834.2
ADD3	DOCK9	CDK19	VTI1A	PDCD6
PITPNB	FBXW2	ADGRA3	PRKRA	CEP295
NUDT14	ADO	ALG13	TUT4	MMAA
TMEM181	TMEM134	PRKX	CARD8	ZNF70
SCAMP4	SOS1	SREK1	UBN2	TAMM41
BUD23	CSNK1G3	TFDP2	TRERF1	ADNP-AS1
TMEM205	MGAT4A	ZNF275	AC007406.4	ZNF701
GOLGA3	RRP7A	AQR	AC009237.15	EVC2
KTN1	RLIM	TASP1	NAA16	KIAA1958
DEDD2	CDK12	CTU2	ARNTL	ZNF8
VPS51	PANK3	SPATA13	WDR7	AKAP7
KBTBD2	TMF1	AGTPBP1	TLE4	RNASEH2B
TPR	RP2	C16orf72	ZNF226	TPCN2
PDCD4	HECA	EP400	MID2	AC139795.2
CELF1	GALC	MRE11	SAMD12	PCSK7
SEPTIN11	MAP3K7	ODF2L	PTPN21	MTHFD2L
CCAR1	ANKRD50	CBR4	SORBS1	BMP2K
HPS1	LEMD3	DOCK5	ZNF440	AL138756.1
TUSC2	JMJD1C	SNORD19C	AC055811.4	NBPF1
MBD2	AIMP1	NOL8	UVSSA	MCM9
ITPRID2	TRIM2	CMAHP	CEP44	MTRF1L
SLC27A4	PKD2	MIDEAS	CLK4	MBLAC2
DDX54	STAU2	UHRF2	HACD4	ZNF570
TSR2	MCRIP2	GSK3B	SLC44A5	FAM221A
HECTD1	ARHGAP21	PPM1B	SLC9A6	AL513550.1
C16orf91	ARIH2	RNU6-1016P	RAD18	THAP9
SLFN5	LINC00342	ZNF138	HMGN2P5	ZBTB8A
SERTAD1	ZC3H13	SRBD1	LYRM7	AL139099.3
SMIM29	AC097448.1	SOCS5	POU2F1	FANCC
RBM12	PIK3CB	C12orf4	FARP1	BICD1
H2AJ	B3GALT4	RSBN1	TTC30A	IL7
AFTPH	MCAT	UVRAG	CCSAP	FAN1
BLOC1S4	TOR1AIP2	RNGTT	PTPN14	ZNF836

DCAF6	UBE4A	TYW3	ZSCAN30	SLC35D2
MSRB2	TRMT1L	ARMC8	PMS2	CDKL5
SHOC2	EXOC1	DYNC2LI1	AHI1	ZSCAN22
ATF6	USP32	NF1	TRAPPC10	C14orf28
TMED5	PRRG2	SNORA65	HCFC2	AL136295.6
STAP2	ABCB10	FBXO11	RASGEF1B	TYW5
B4GALT2	ZNF33A	ADAM17	ZFHX3	CHROMR
ARL2	ROCK1	BOD1L1	AZI2	MIR4453HG
MMGT1	NUP58	SLMAP	ZNF616	AC008124.1
LGR4	AKAP10	TAF1B	MTERF1	RRN3P3
FNDC3A	SLC5A3	PHF14	SCFD1	C15orf40
APOL6	BIRC6	TRANK1	VPS13A	KLC1
DYNC1LI2	PHF20	BTBD7	PHKA2	ERCC6L2
BCLAF1	AASDHPPT	PLEKHA5	AC015813.1	ZNF813
MAPK14	CNST	TMEM131L	VPS53	MEIS2
SEC63	FGD6	VPS13D	KIZ	GUSBP1
MAN1B1	C3orf38	PPP2R3A	OSGEPL1	RAPGEF6
POGK	CASP8	ZMYM5	IMMP2L	THAP2
SPTLC1	QKI	CNEP1R1	GPRIN3	XYLB
KPNA6	PPWD1	NIPA1	RNPC3	AC096992.2
SRRM1	MAP4K5	MBTD1	GNPDA2	AFAP1
FAM20B	OTUD1	COG6	KIAA0895	KRIT1
RFXANK	SPEN	DLG1	STXBP5	DNAJC27
ZNF358	SHLD2	TIGD2	TBK1	EFCAB7
TMEM33	SAC3D1	ANKRD46	KANSL1L	MRPL45P2
RNF113A	ASXL1	KIAA1109	HMCN1	AC135721.1
SWAP70	PAN3	ORC4	N4BP2L1	MICOS10
NSMCE1	NBAS	ZNF480	GTPBP8	AP002840.2
G3BP2	SMARCAD1	ZBTB6	AC108449.2	TMOD2
URM1	TAF2	TCAIM	ZNF426	ZNF610
SURF2	C2CD5	ZDHHC13	TAF1A	ZNF619
TMEM245	CLASP1	FAM122A	SLC25A30	NRIP2
FAT1	PREPL	MKLN1	MFAP3	AC005332.4
NUP98	RSBN1L	NDUFAF7	GTF2I	ZNF433
WAC	TASOR2	ZNF740	FTO	SRP19
FNDC3B	ZHX1	OTUD7B	FAM126B	DTWD1
ENAH	LIFR	NEBL	METTL4	ZNF17
CCDC71	CARD19	IPP	ANKRD44	ADAL
HECTD3	MTOR	HIBCH	ATXN3	AC020915.2
DDX49	LRRC61	SNTB2	PPARA	FKTN
TTC3	TADA1	ETFDH	TRAF6	L3MBTL4
LRRFIP1	BAD	CYP7B1	DNAH5	LSM11
SLC44A1	ATG14	SRFBP1	MIR573	KAT6B

CRIM1	OTUD4	LIG4	CIBAR1	ZNF620
UBTD2	MRPL4	AMPD3	AC147067.1	KIAA0586
TSSC4	PCYT2	SUPT20H	EEF2KMT	ZKSCAN2
DCTN4	SENP6	RPUSD2	SYNJ1	TUBGCP4
USP9X	PBRM1	RIF1	ARHGAP19	ATXN7L1
EMC10	NEPRO	MAST4	DNAL1	ZNF175
ODR4	REX1BD	WDR37	ICE2	TCEANC2
NBDY	PSMD6	REPS1	TSC1	CCNK
ARFIP1	COX11	FAM117B	CDC14B	IBA57
RHOBTB3	WDSUB1	PIKFYVE	ZNF562	NRDE2
RPE	NEK9	ZDHHC17	RBM45	ZFP28
ZNF281	TCERG1	FPGT	REV3L	ZNF419
NFKBIB	UBE2W	SNORD14E	SLFN12	
CLDN12	FKBPL	GOSR2	ZFYVE16	
BAZ1A	PTCD3	POLR2M	LYST	

Table S5. The corresponding coefficients of 16 model genes

Gene	Coef
PKM	0.001729711
S100A16	0.000969081
RRAS	0.000852838
TUBA4A	0.000299351
PKP3	0.001901506
KCTD12	-0.006773274
LPGAT1	0.023038344
ITPRID2	0.00109672
MZT2A	0.00668661
LIFR	-0.000874168
PTPRM	0.008020131
LATS2	0.004912594
PDIK1L	-0.046445765
GORAB	-0.018922196
PCDH7	0.020683155
CPED1	-0.005776393
